# Supplementary figures and images for: Disintegration promotes protospacer integration by the Cas1-Cas2 complex
Source: eLife. 2021 Aug 26;10:e65763. doi: 10.7554/eLife.65763 (PMC8390005; doi:10.7554/eLife.65763)

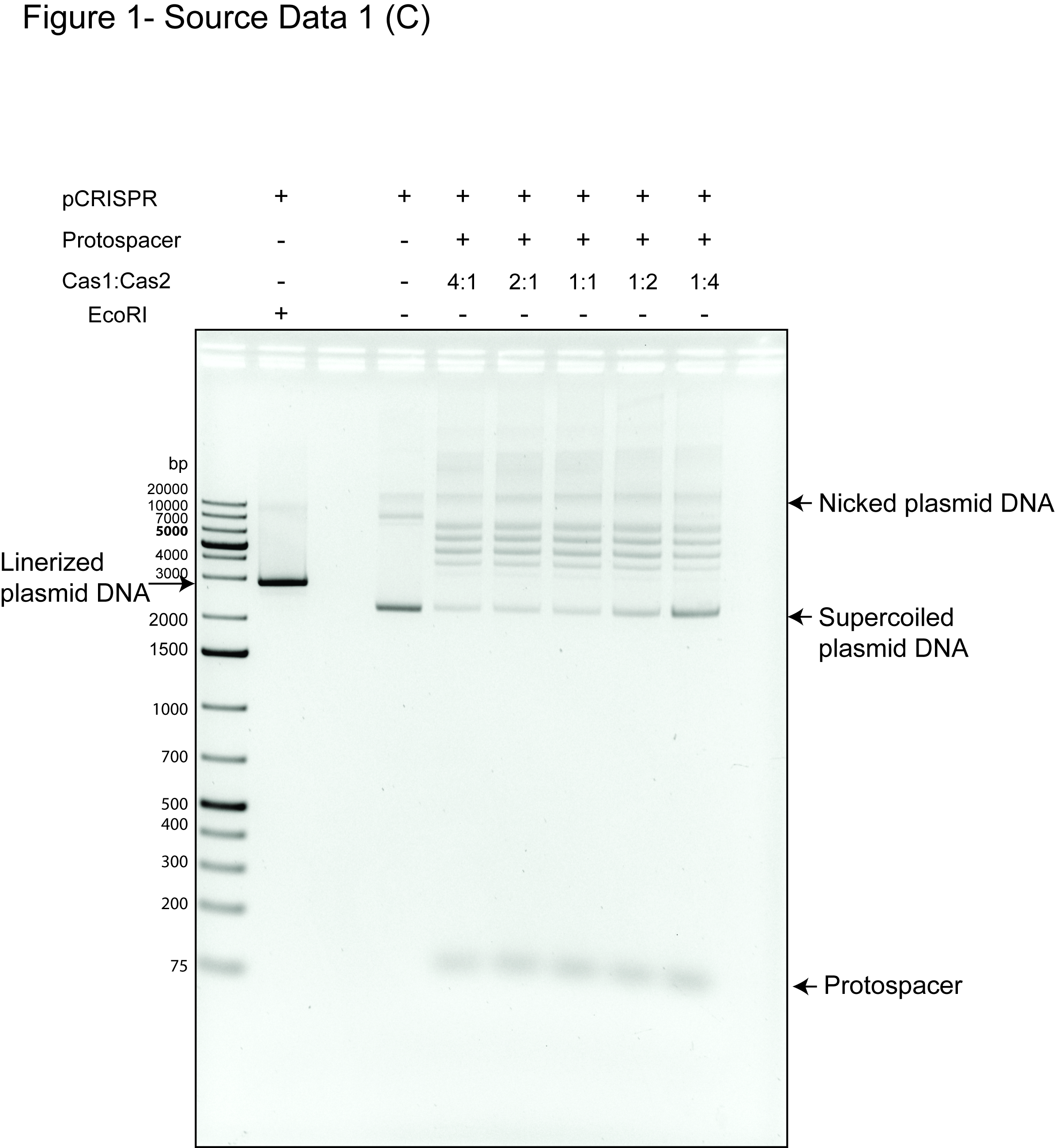

Supplement: Source data 1. [file elife-65763-data1.zip › CRISPR paper-Source Data-1/Figure 1- Source Data 1 (C).tif]

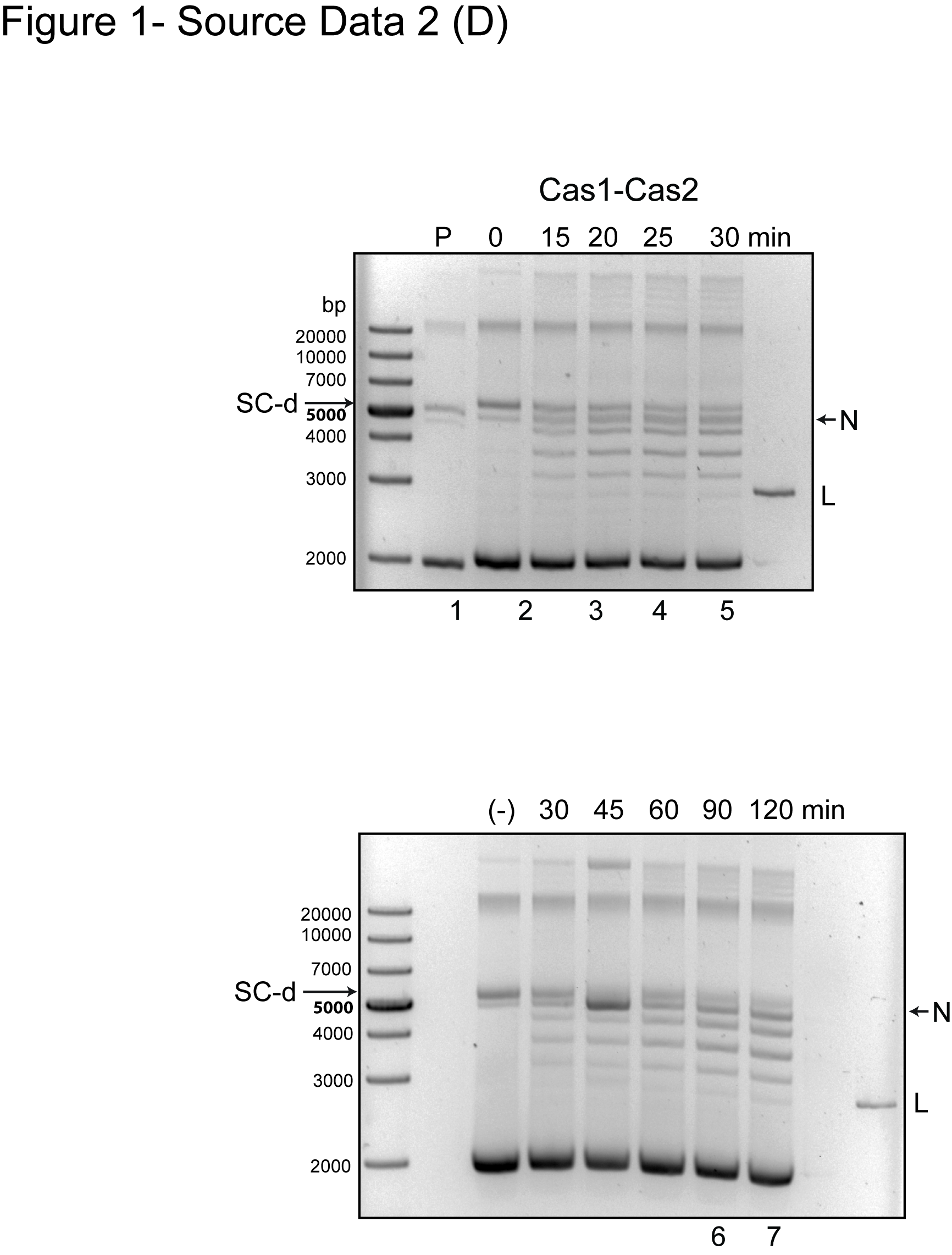

Supplement: Source data 1. [file elife-65763-data1.zip › CRISPR paper-Source Data-1/Figure 1- Source Data 2 (D).tif]

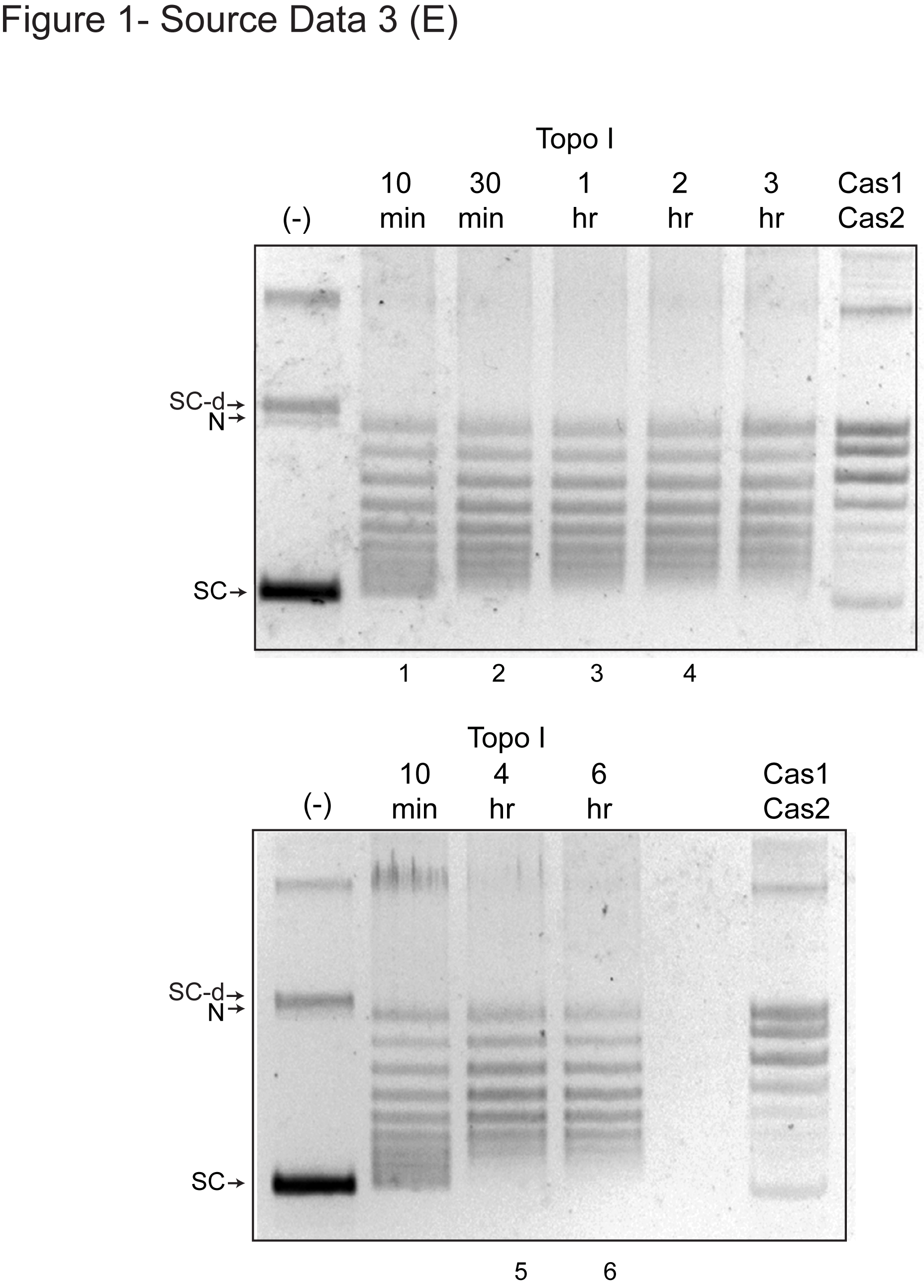

Supplement: Source data 1. [file elife-65763-data1.zip › CRISPR paper-Source Data-1/Figure 1- Source Data 3 (E).tif]

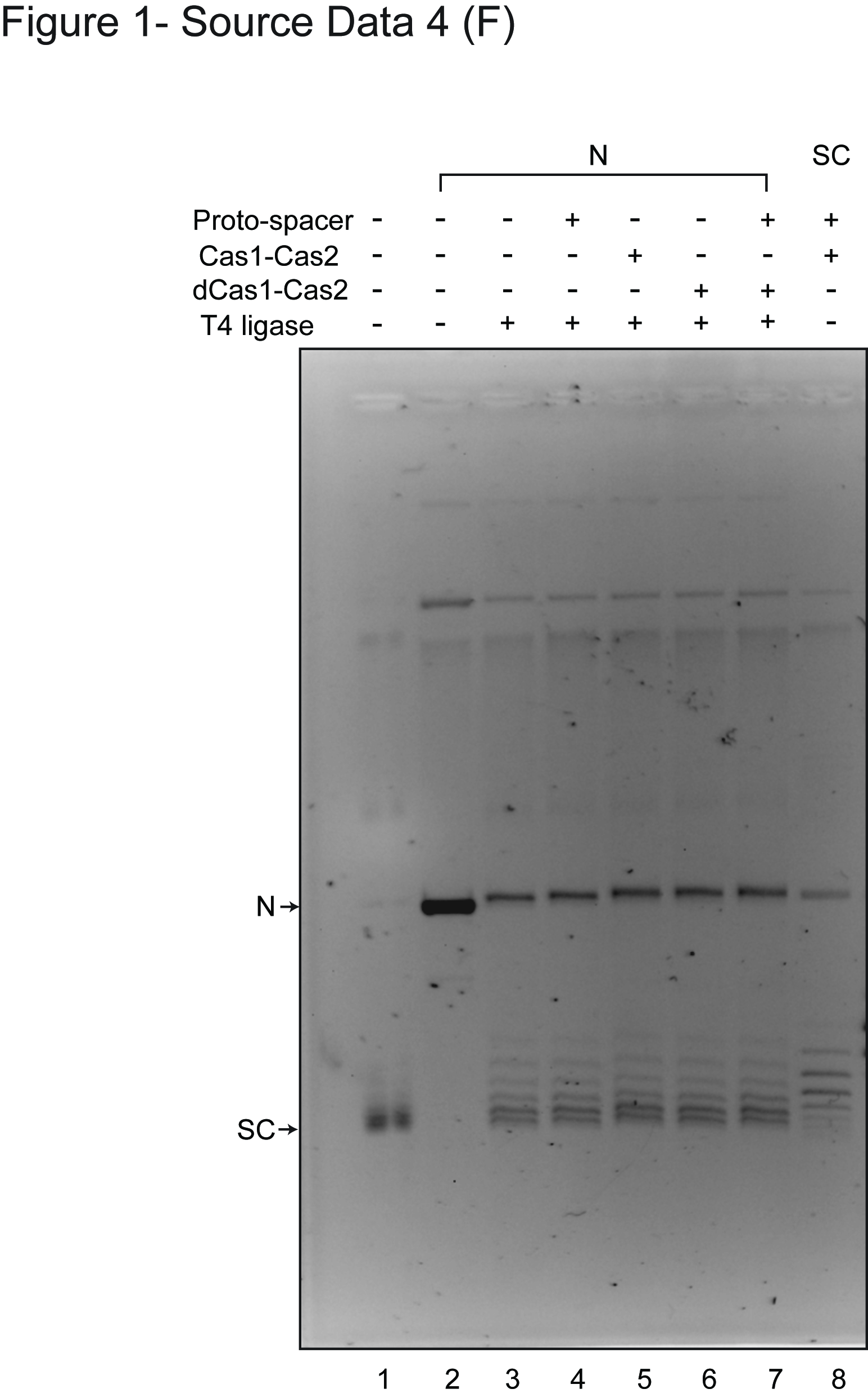

Supplement: Source data 1. [file elife-65763-data1.zip › CRISPR paper-Source Data-1/Figure 1- Source Data 4 (F).tif]

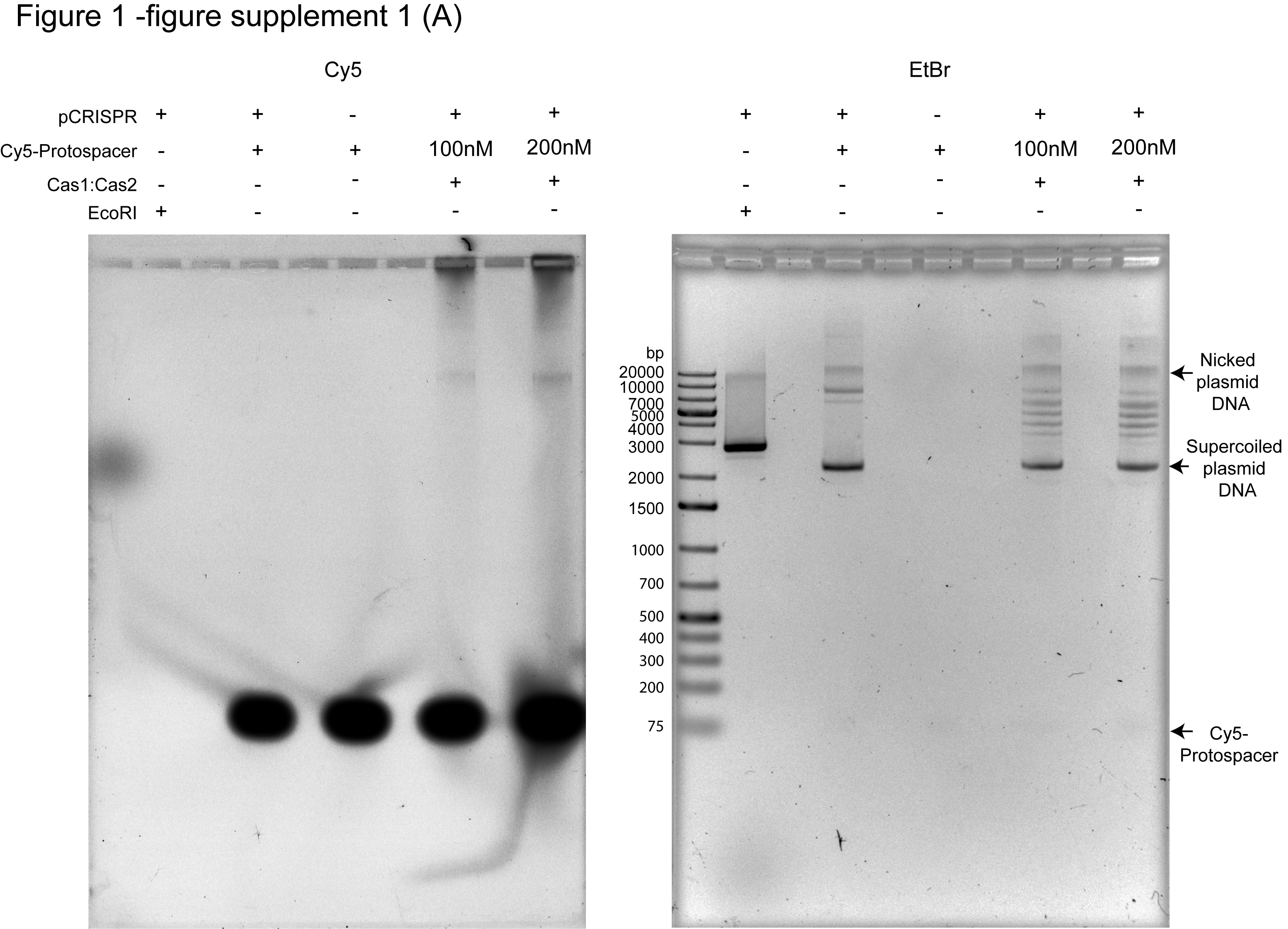

Supplement: Source data 1. [file elife-65763-data1.zip › CRISPR paper-Source Data-1/Figure 1-figure supplement 1 Source Data 1 (A).tif]

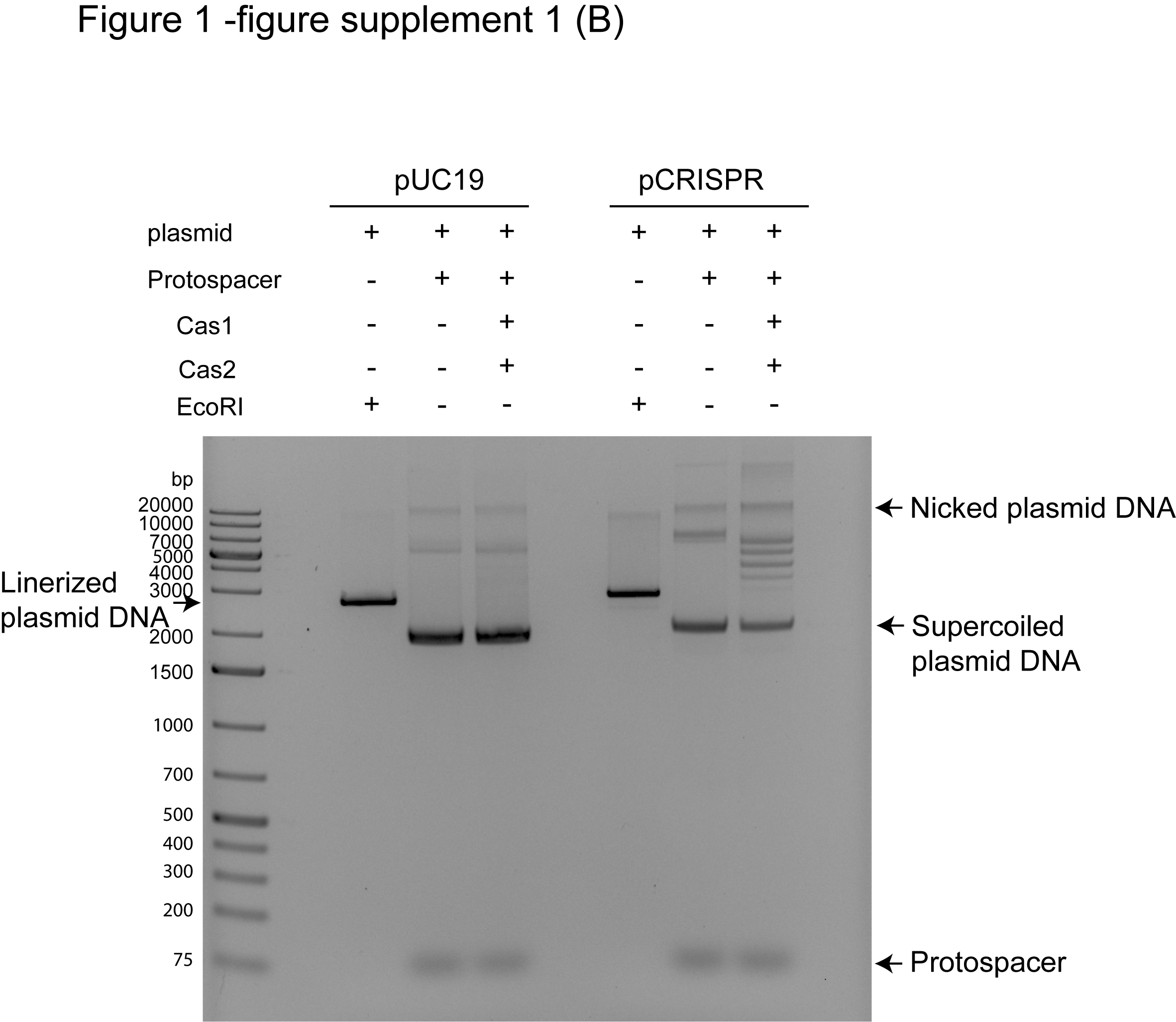

Supplement: Source data 1. [file elife-65763-data1.zip › CRISPR paper-Source Data-1/Figure 1-figure supplement 1 Source Data 2 (B).tif]

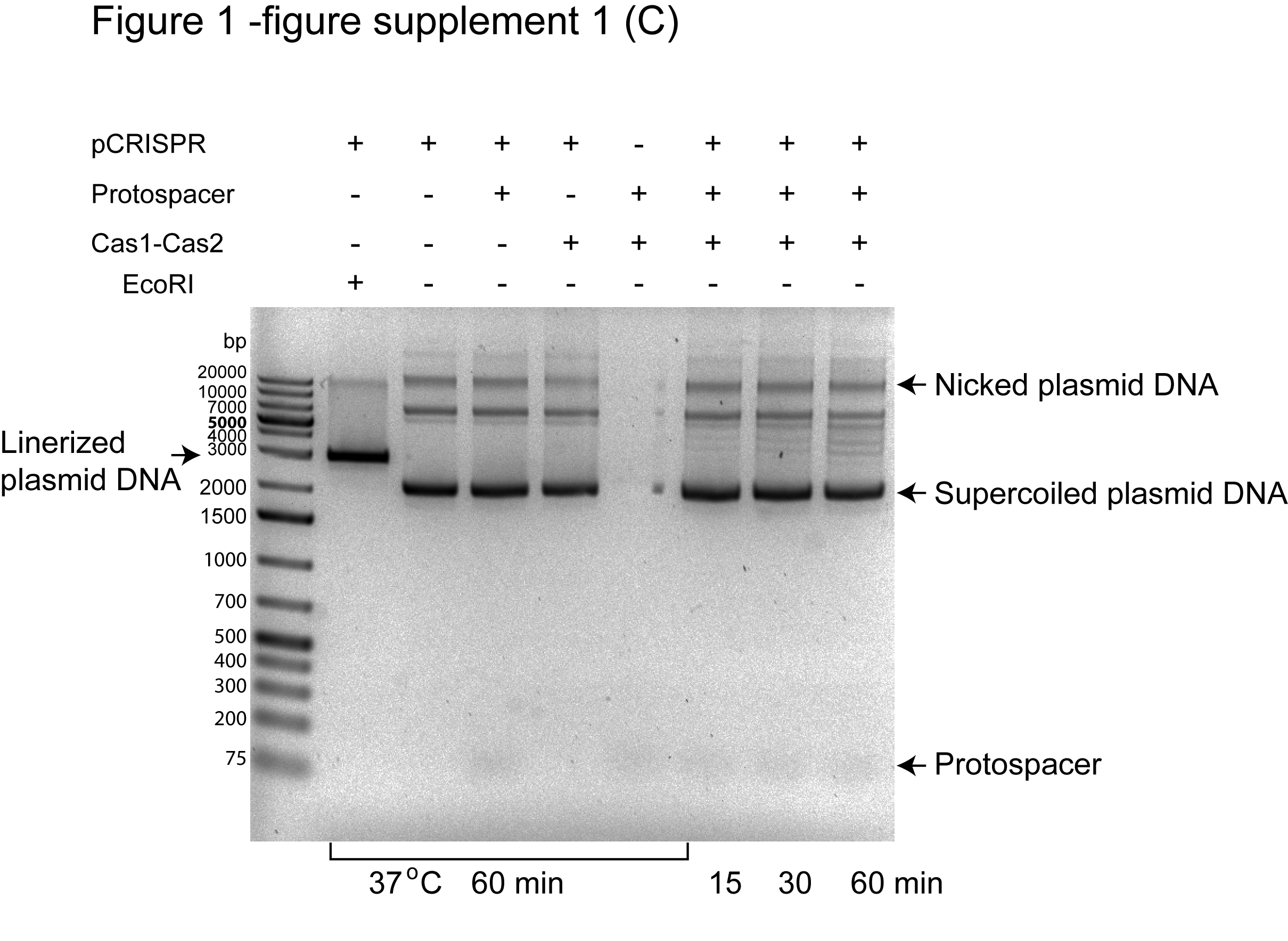

Supplement: Source data 1. [file elife-65763-data1.zip › CRISPR paper-Source Data-1/Figure 1-figure supplement 1 Source Data 3 (C).tif]

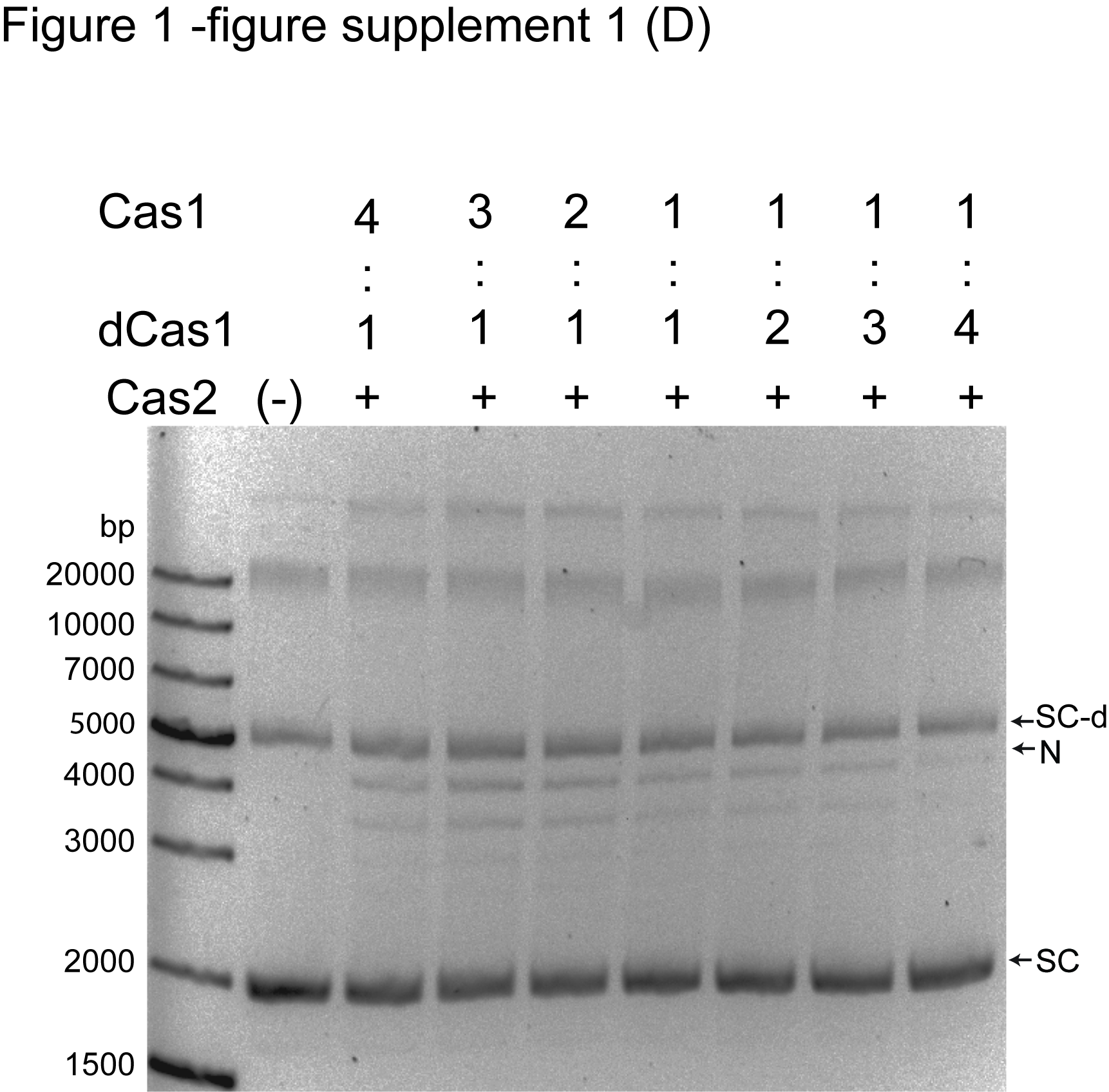

Supplement: Source data 1. [file elife-65763-data1.zip › CRISPR paper-Source Data-1/Figure 1-figure supplement 1 Source Data 4 (D).tif]

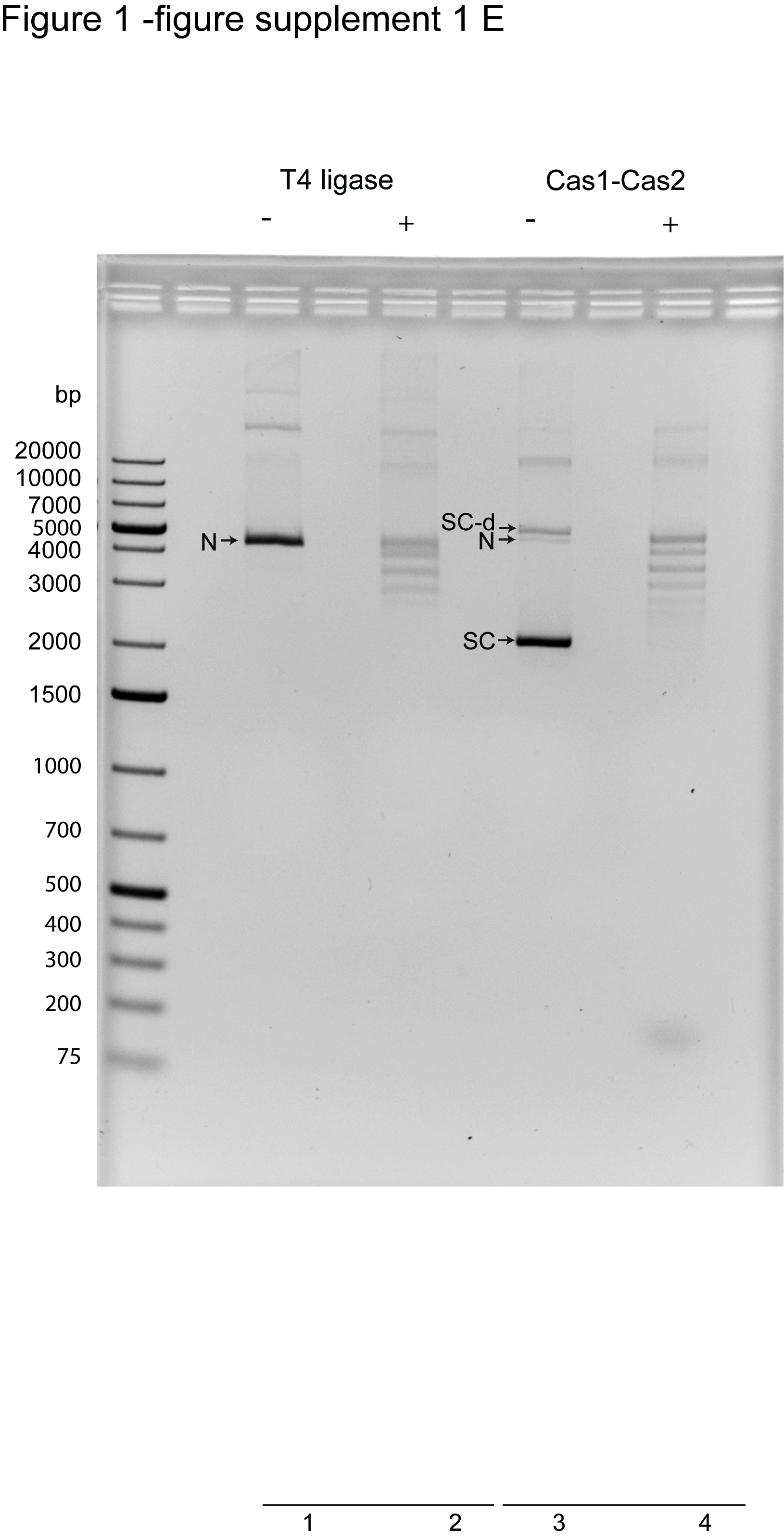

Supplement: Source data 1. [file elife-65763-data1.zip › CRISPR paper-Source Data-1/Figure 1-figure supplement 1 Source Data 5 (E).tif]

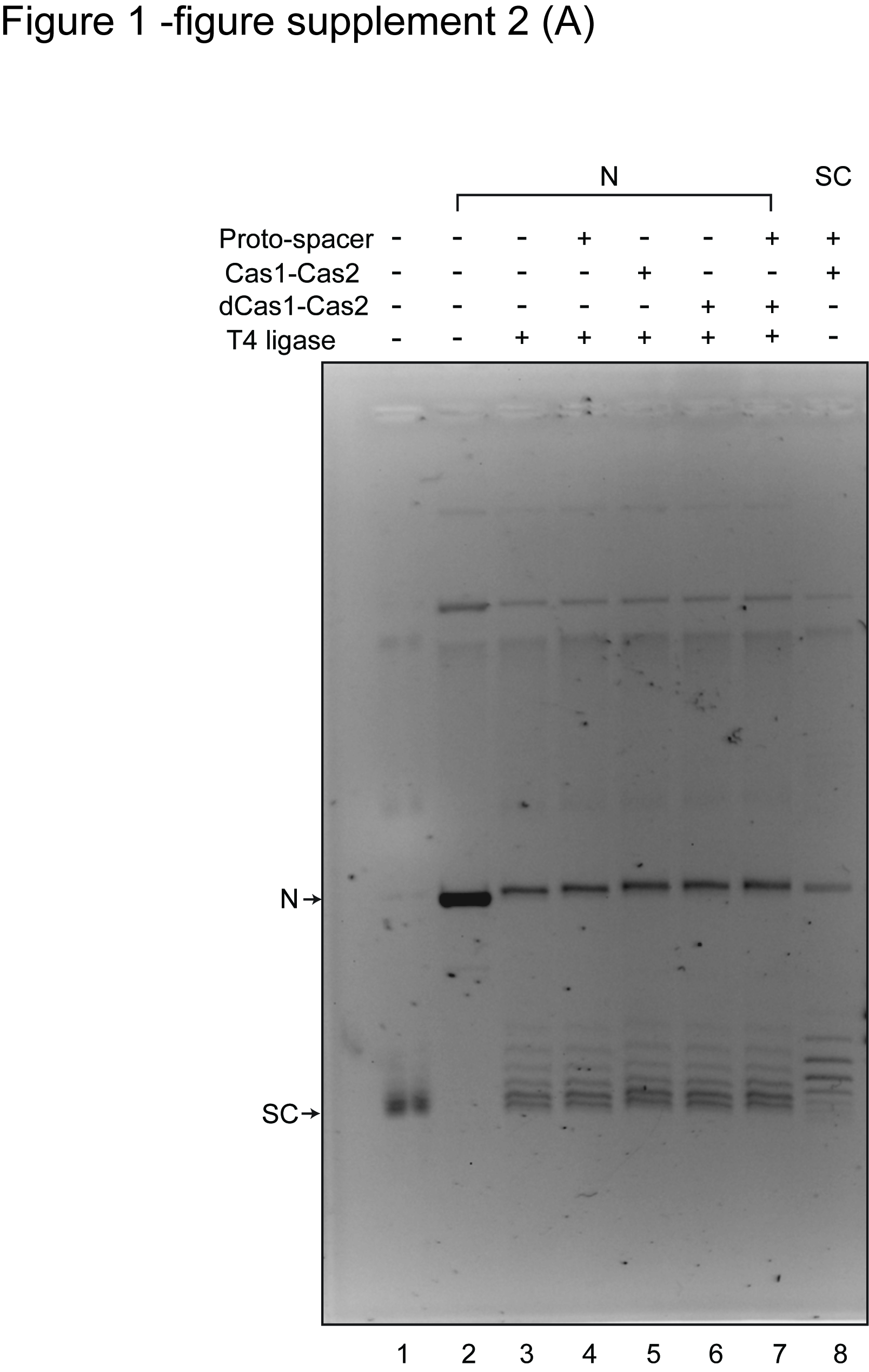

Supplement: Source data 1. [file elife-65763-data1.zip › CRISPR paper-Source Data-1/Figure 1-figure supplement 2 Source Data 1 (A).tif]

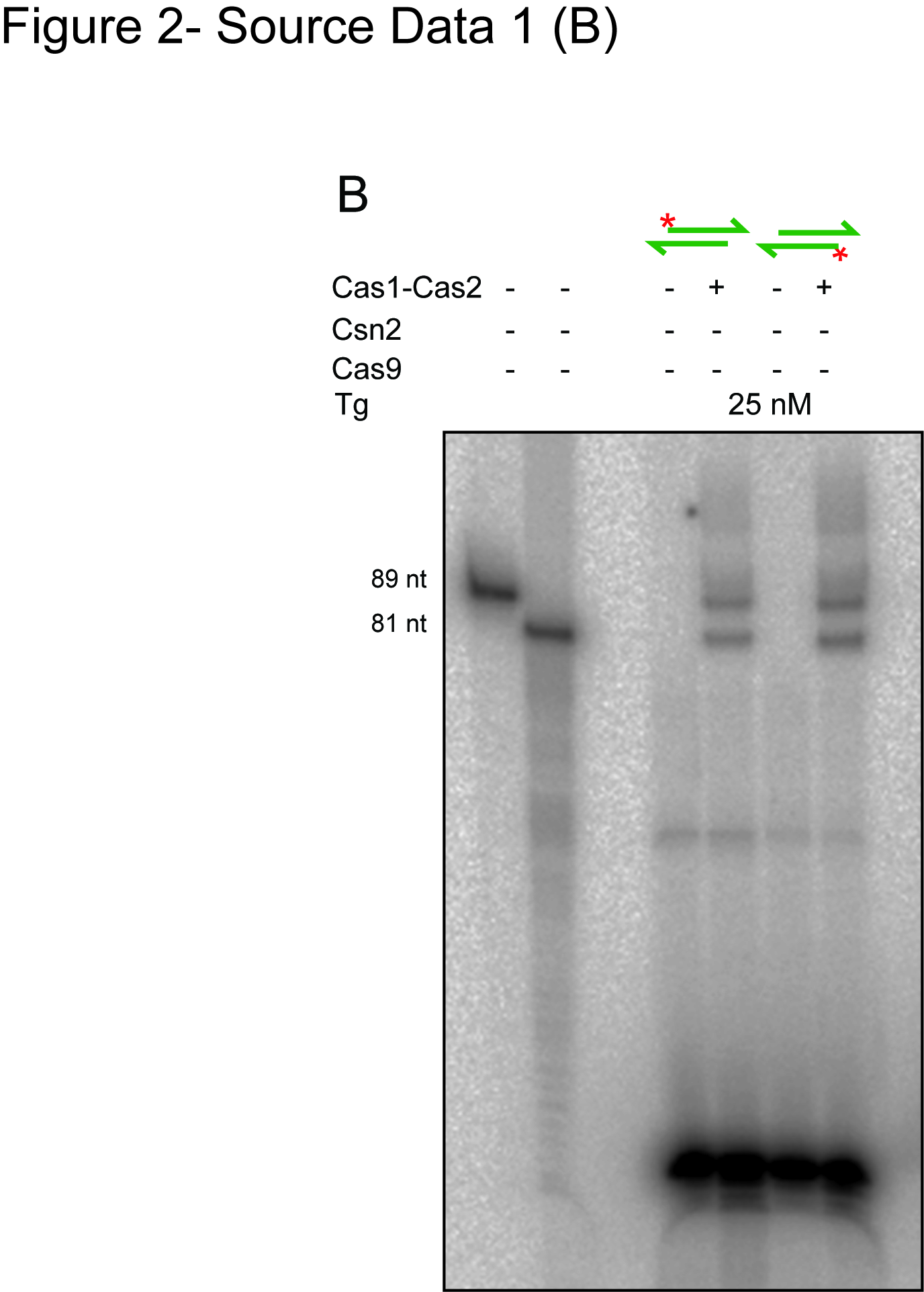

Supplement: Source data 1. [file elife-65763-data1.zip › CRISPR paper-Source Data-1/Figure 2- Source Data 1 (B).tif]

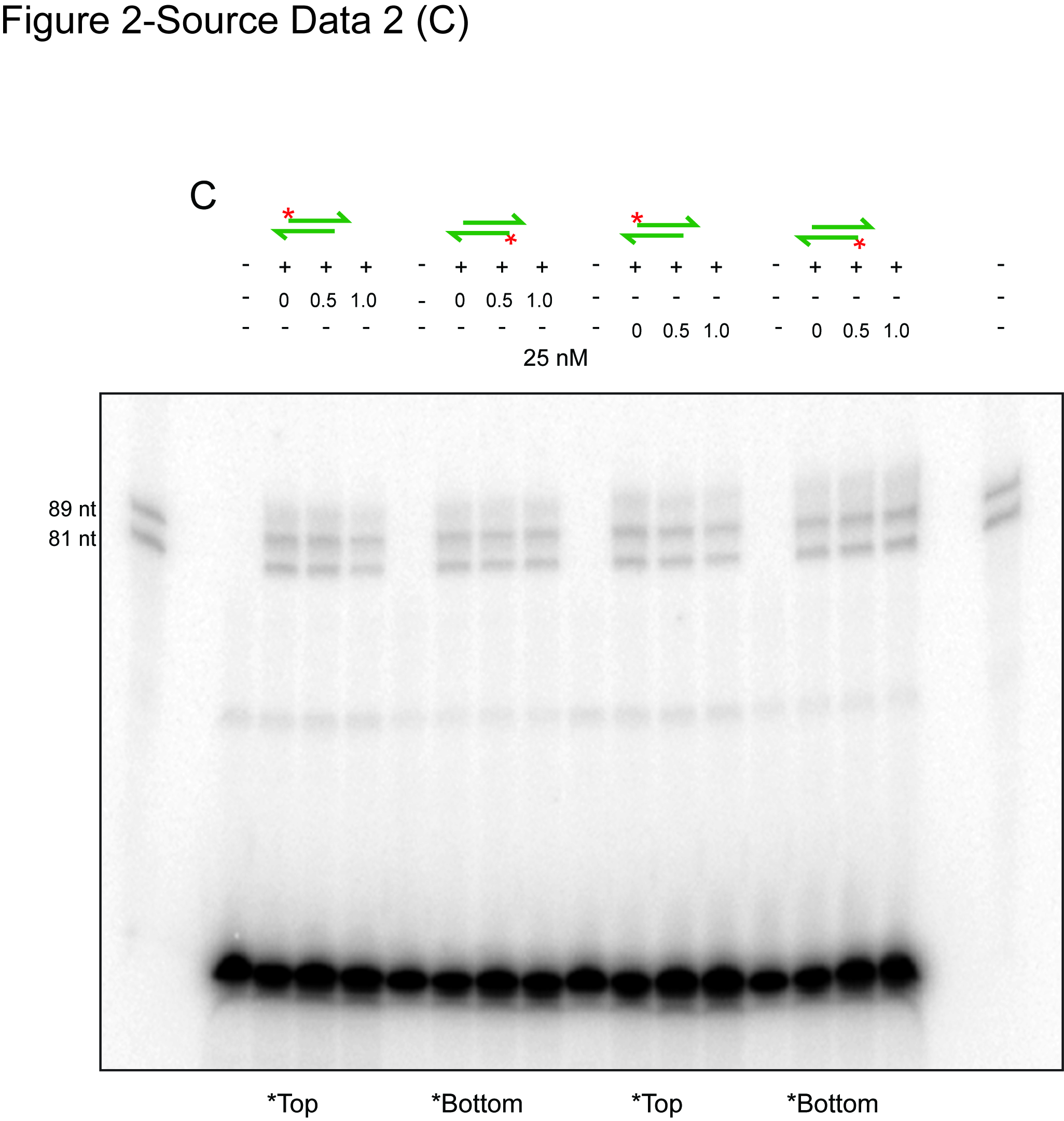

Supplement: Source data 1. [file elife-65763-data1.zip › CRISPR paper-Source Data-1/Figure 2- Source Data 2 (C).tif]

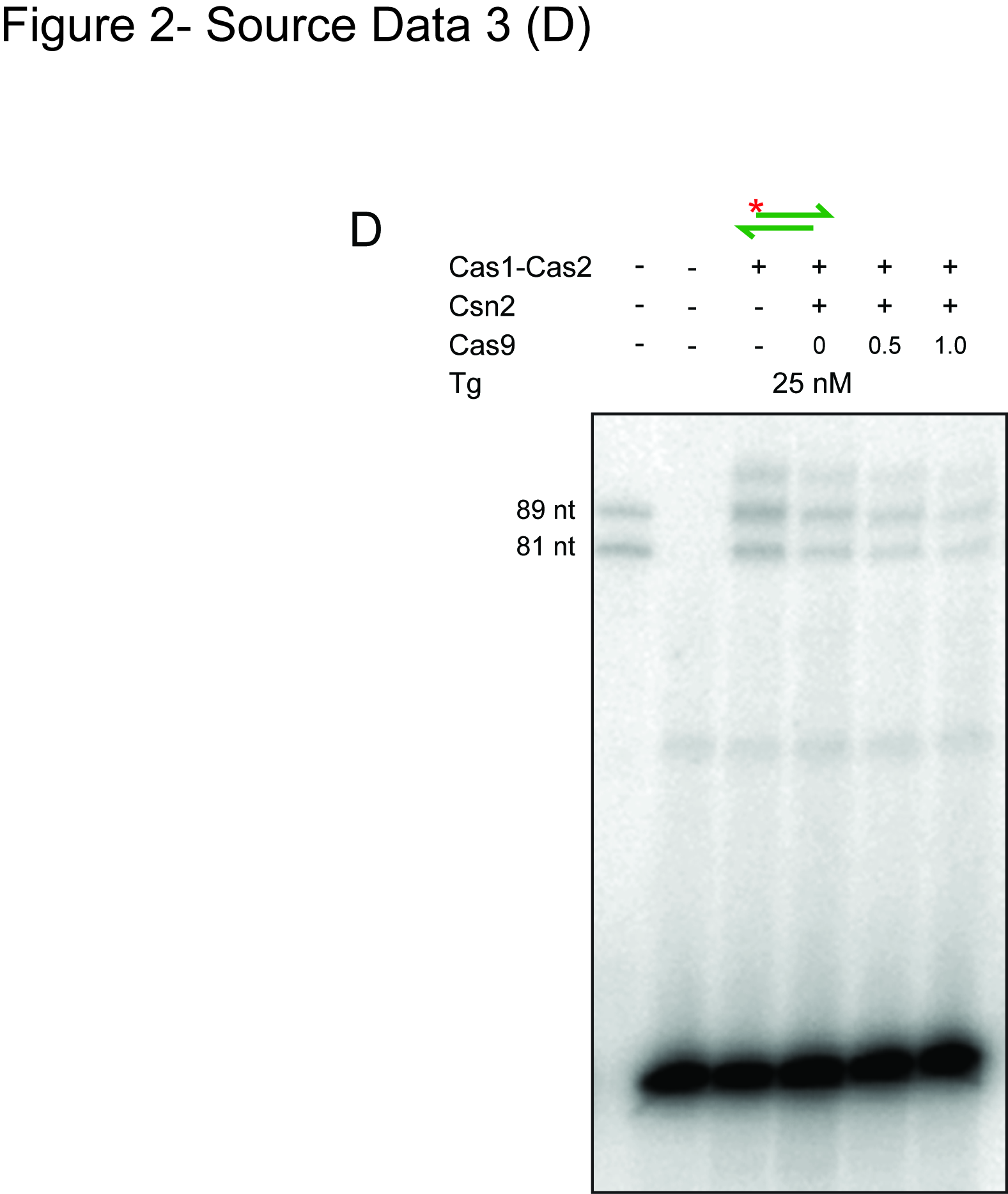

Supplement: Source data 1. [file elife-65763-data1.zip › CRISPR paper-Source Data-1/Figure 2- Source Data 3 (D).tif]

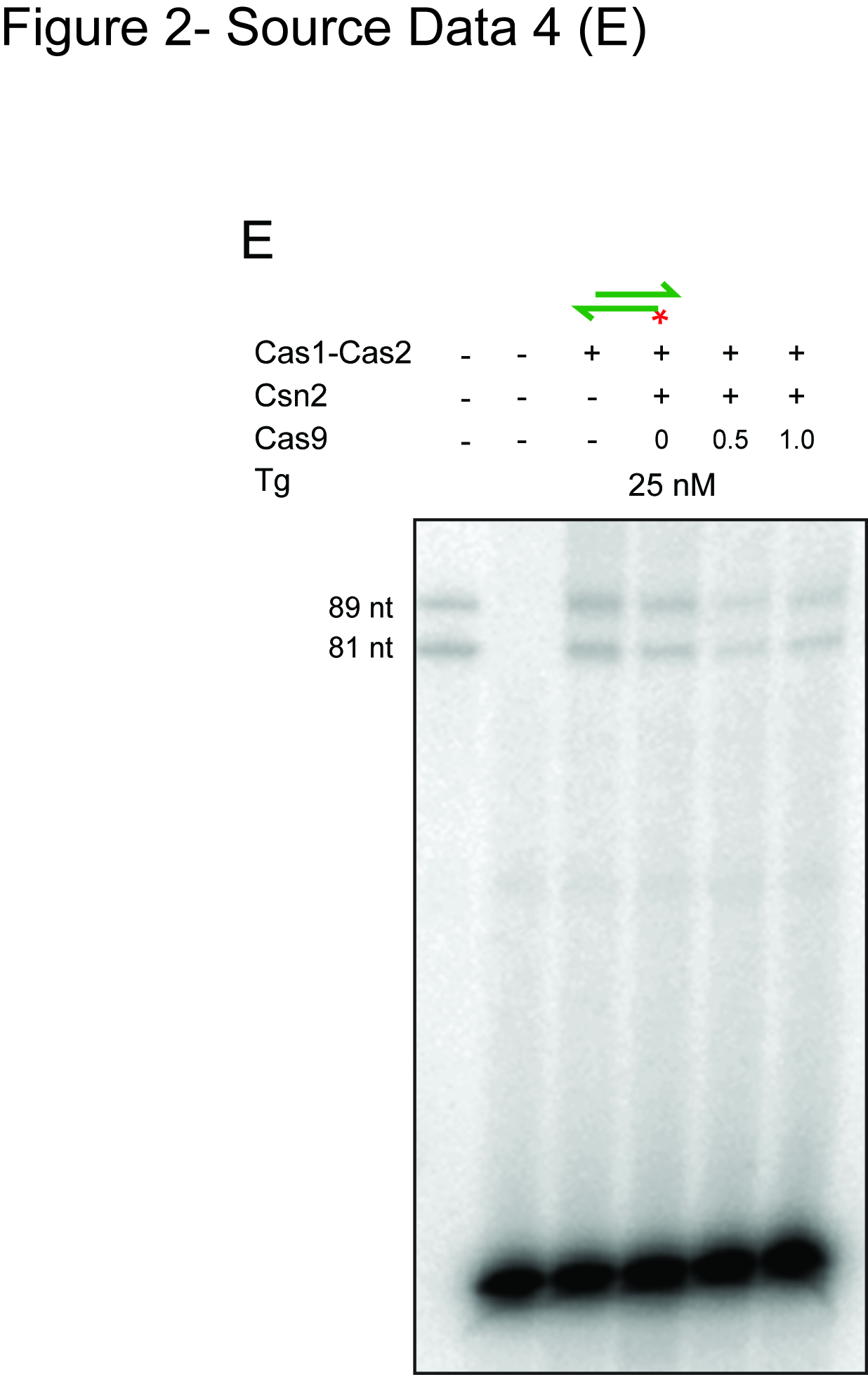

Supplement: Source data 1. [file elife-65763-data1.zip › CRISPR paper-Source Data-1/Figure 2- Source Data 4 (E).tif]

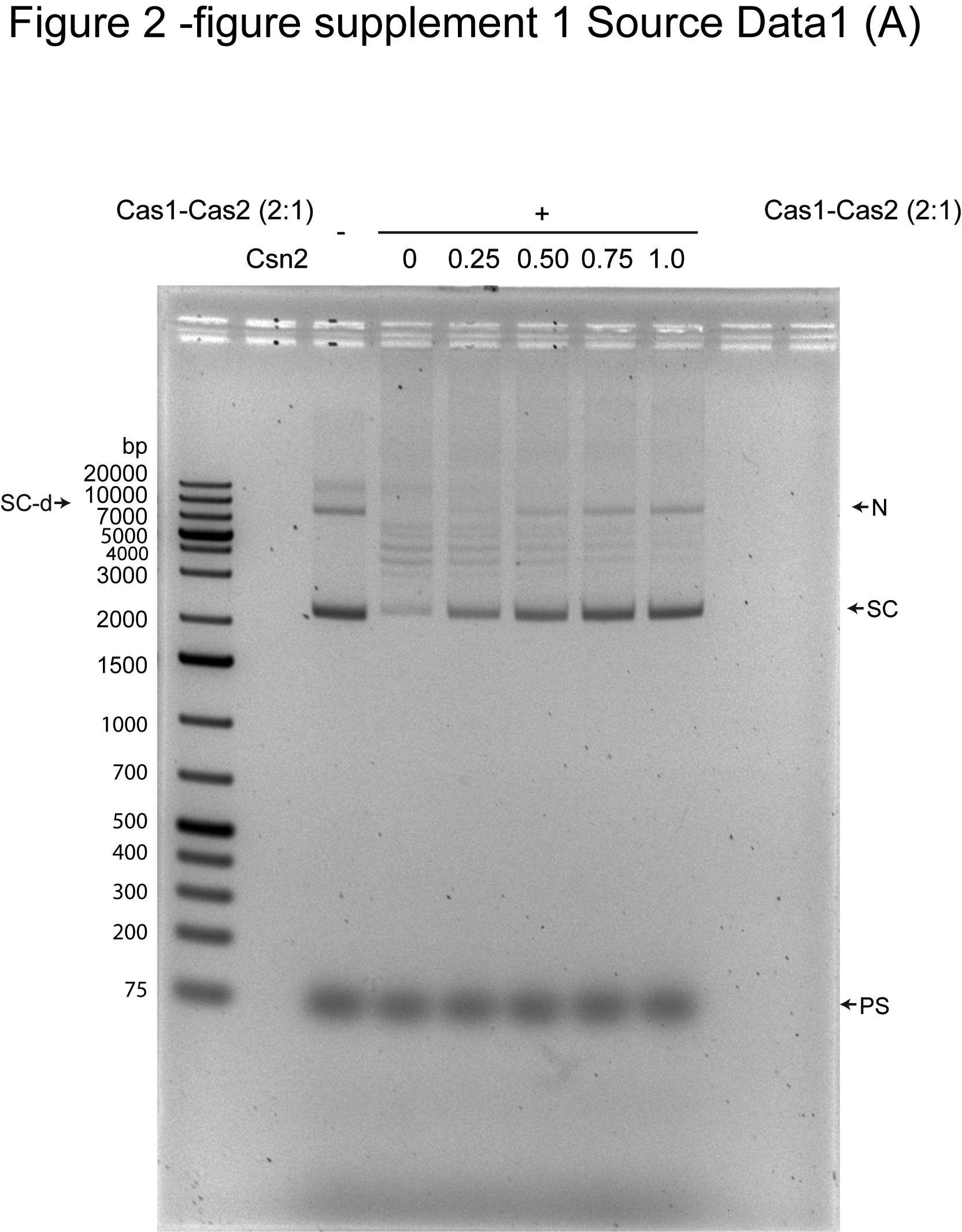

Supplement: Source data 1. [file elife-65763-data1.zip › CRISPR paper-Source Data-1/Figure 2-figure supplement 1 Source Data 1 (A).tif]

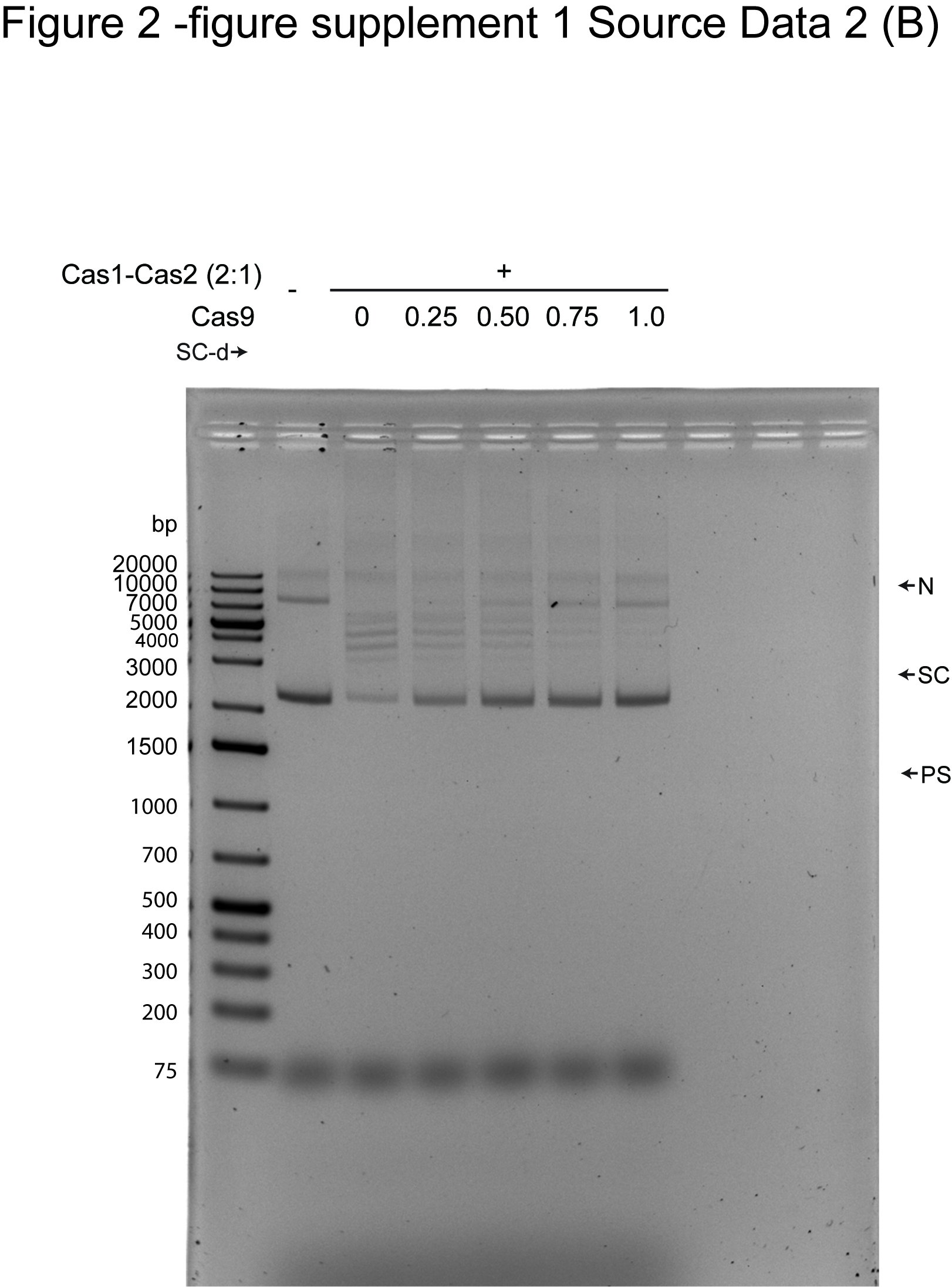

Supplement: Source data 1. [file elife-65763-data1.zip › CRISPR paper-Source Data-1/Figure 2-figure supplement 1 Source Data 2 (B).tif]

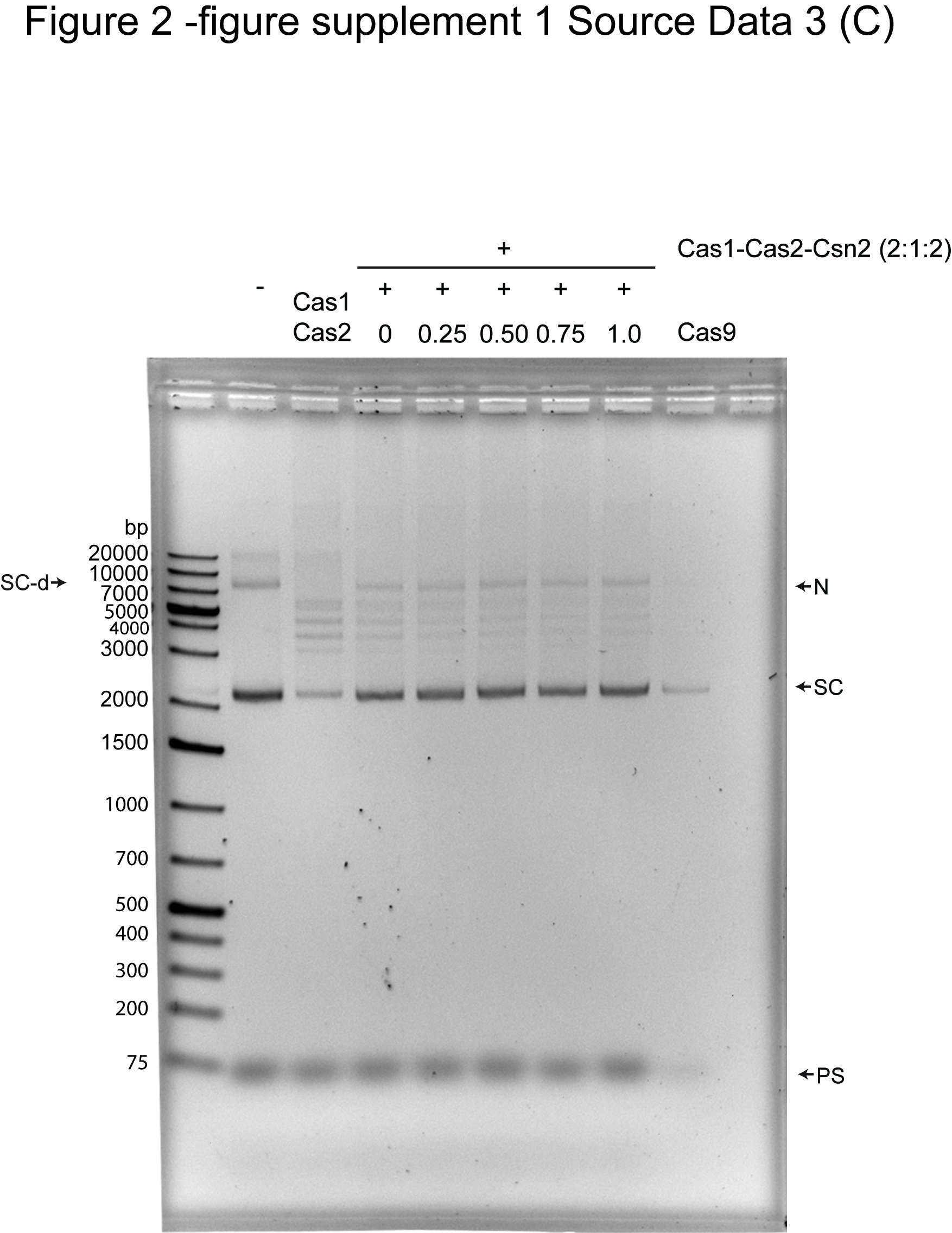

Supplement: Source data 1. [file elife-65763-data1.zip › CRISPR paper-Source Data-1/Figure 2-figure supplement 1 Source Data 3 (C).tif]

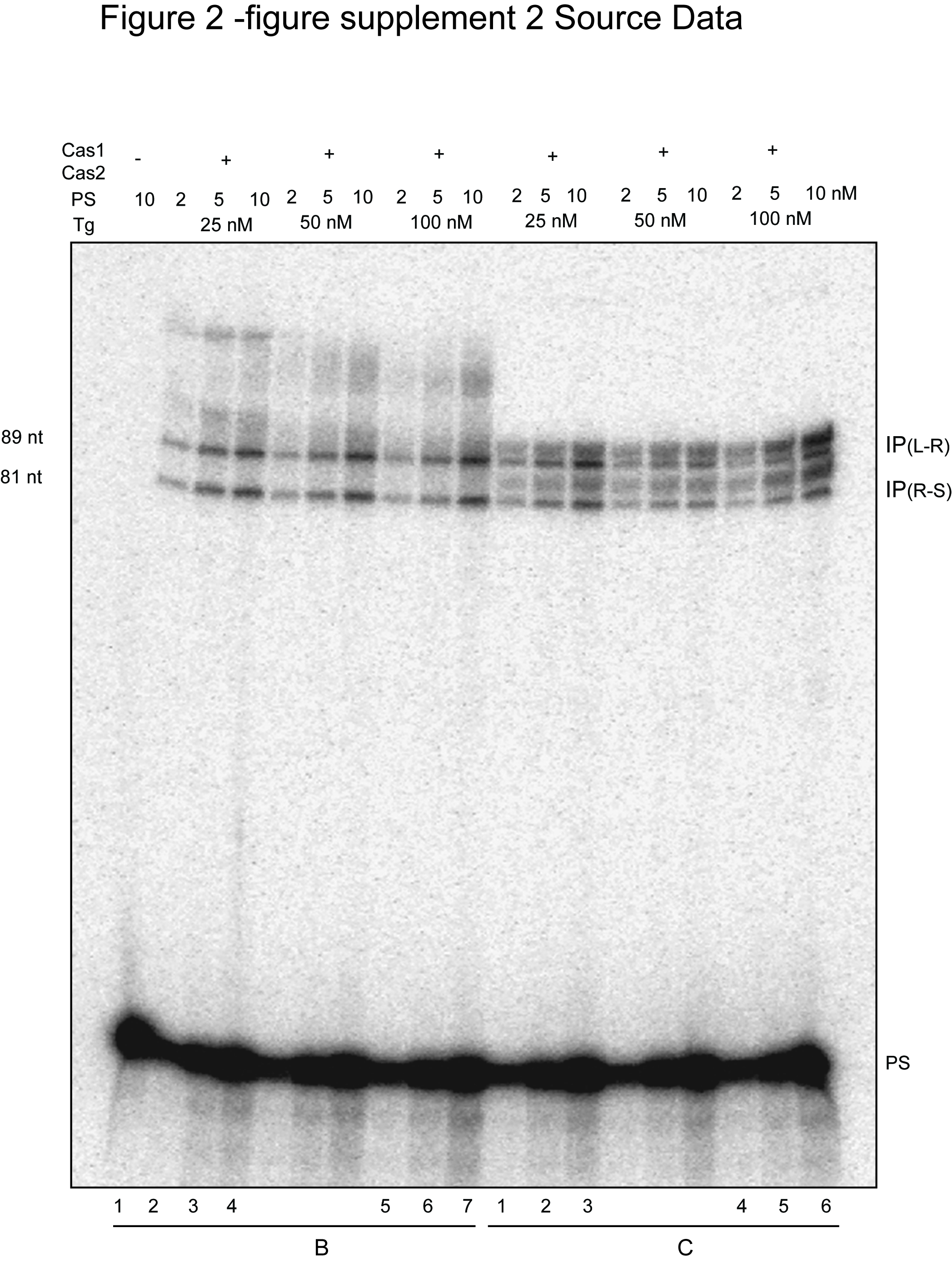

Supplement: Source data 1. [file elife-65763-data1.zip › CRISPR paper-Source Data-1/Figure 2-figure supplement 2 Source Data.tif]

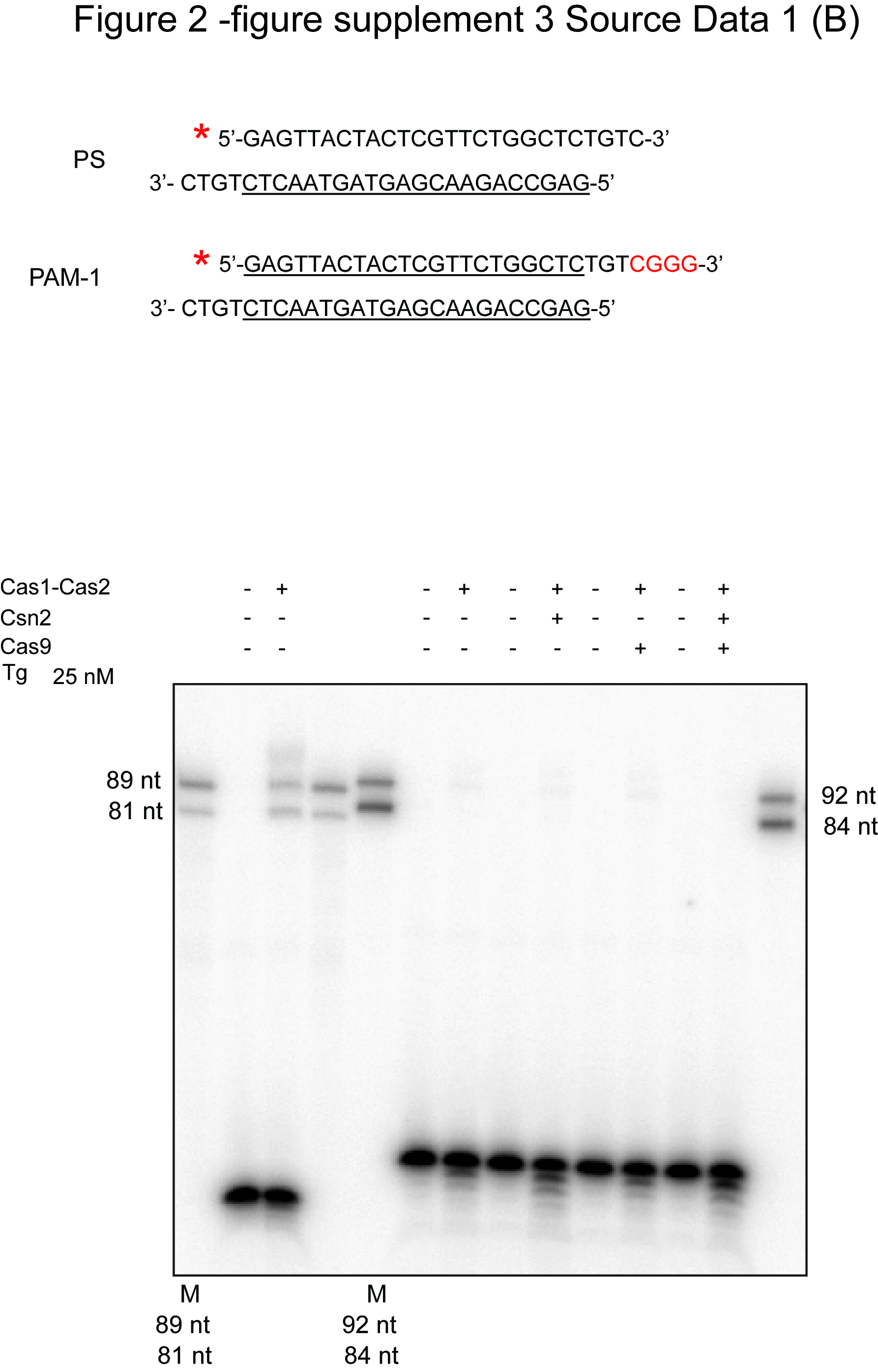

Supplement: Source data 1. [file elife-65763-data1.zip › CRISPR paper-Source Data-1/Figure 2-figure supplement 3 Source Data 1 (B).tif]

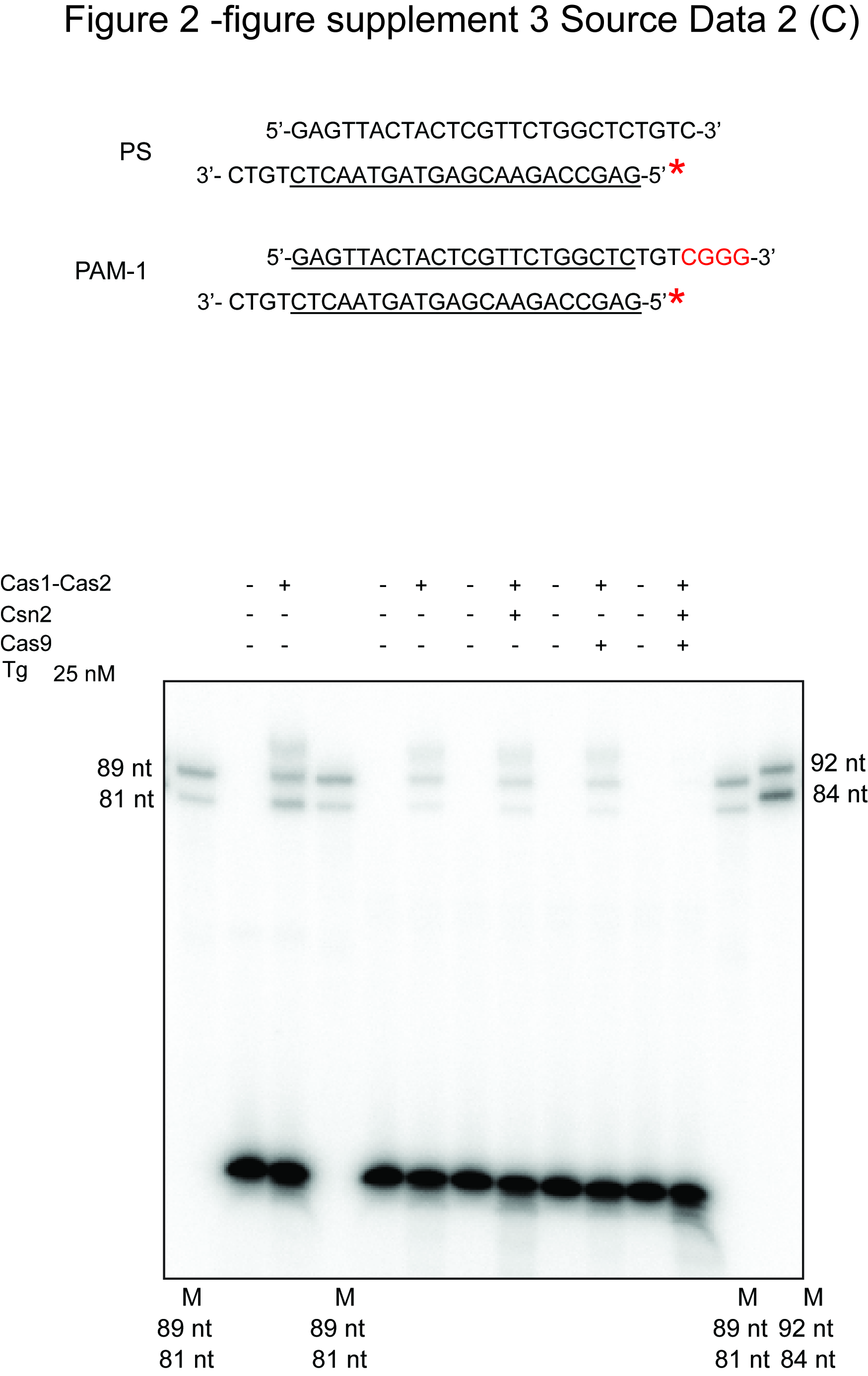

Supplement: Source data 1. [file elife-65763-data1.zip › CRISPR paper-Source Data-1/Figure 2-figure supplement 3 Source Data 2 (C).tif]

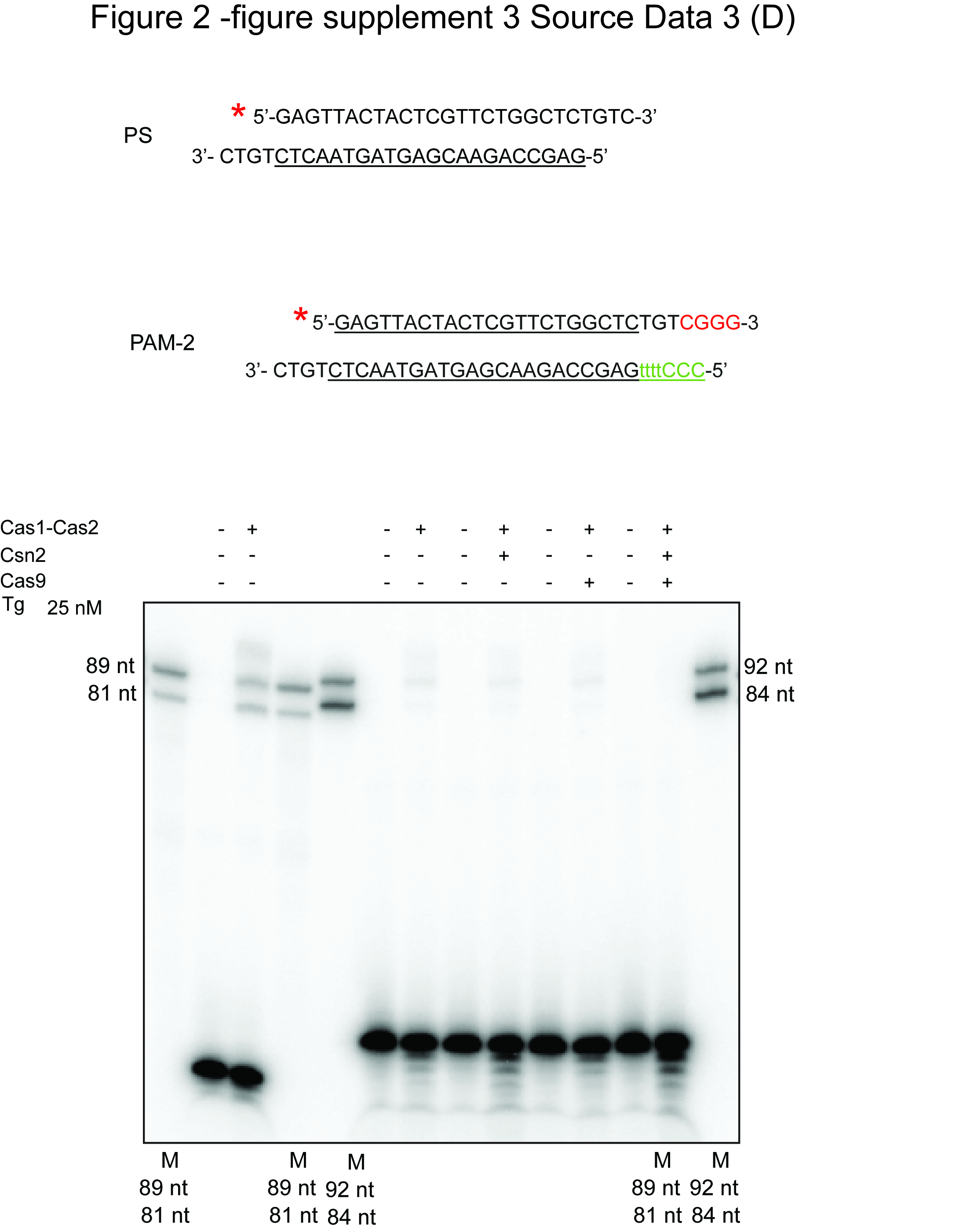

Supplement: Source data 1. [file elife-65763-data1.zip › CRISPR paper-Source Data-1/Figure 2-figure supplement 3 Source Data 3 (D).tif]

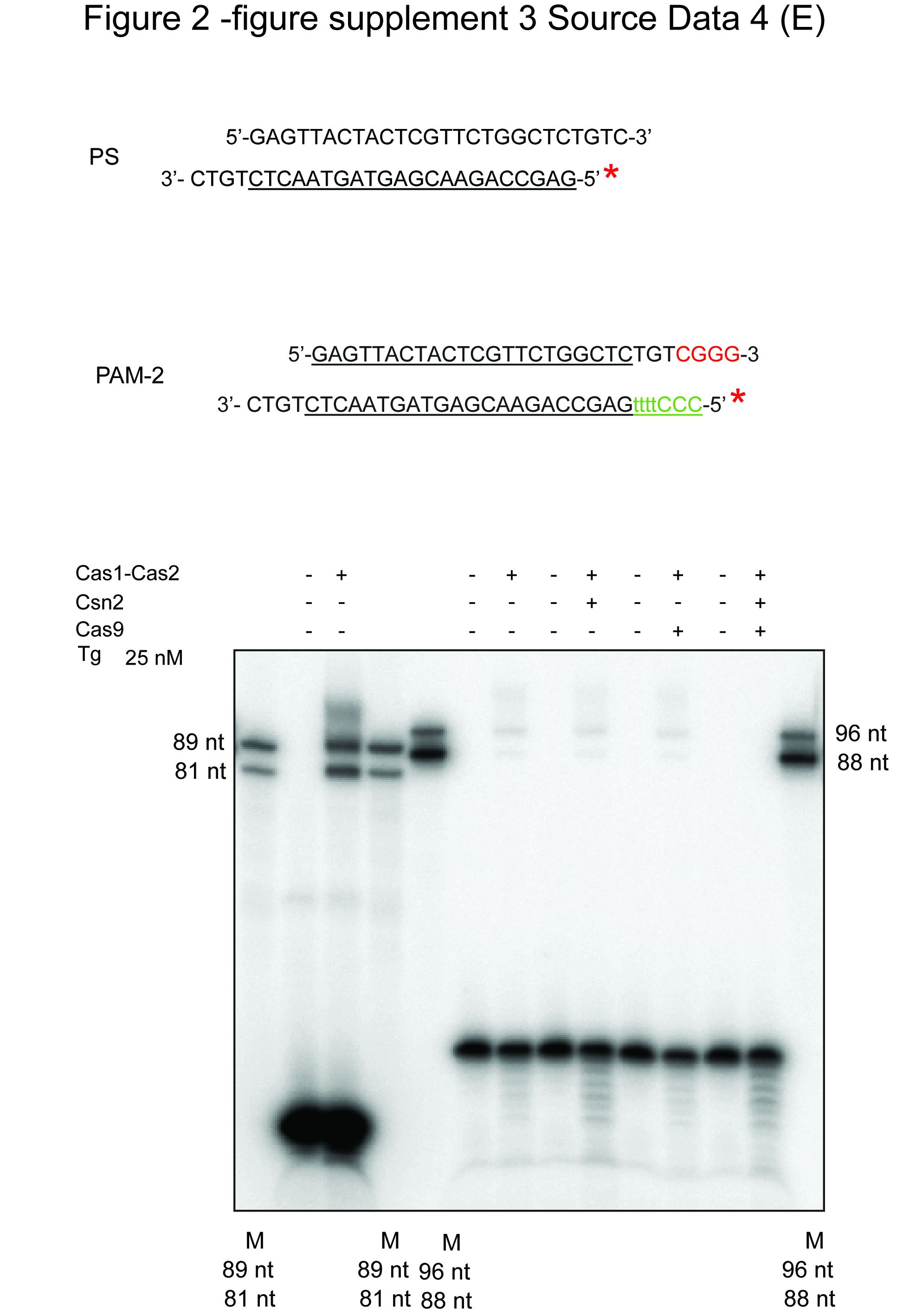

Supplement: Source data 1. [file elife-65763-data1.zip › CRISPR paper-Source Data-1/Figure 2-figure supplement 3 Source Data 4 (E).tif]

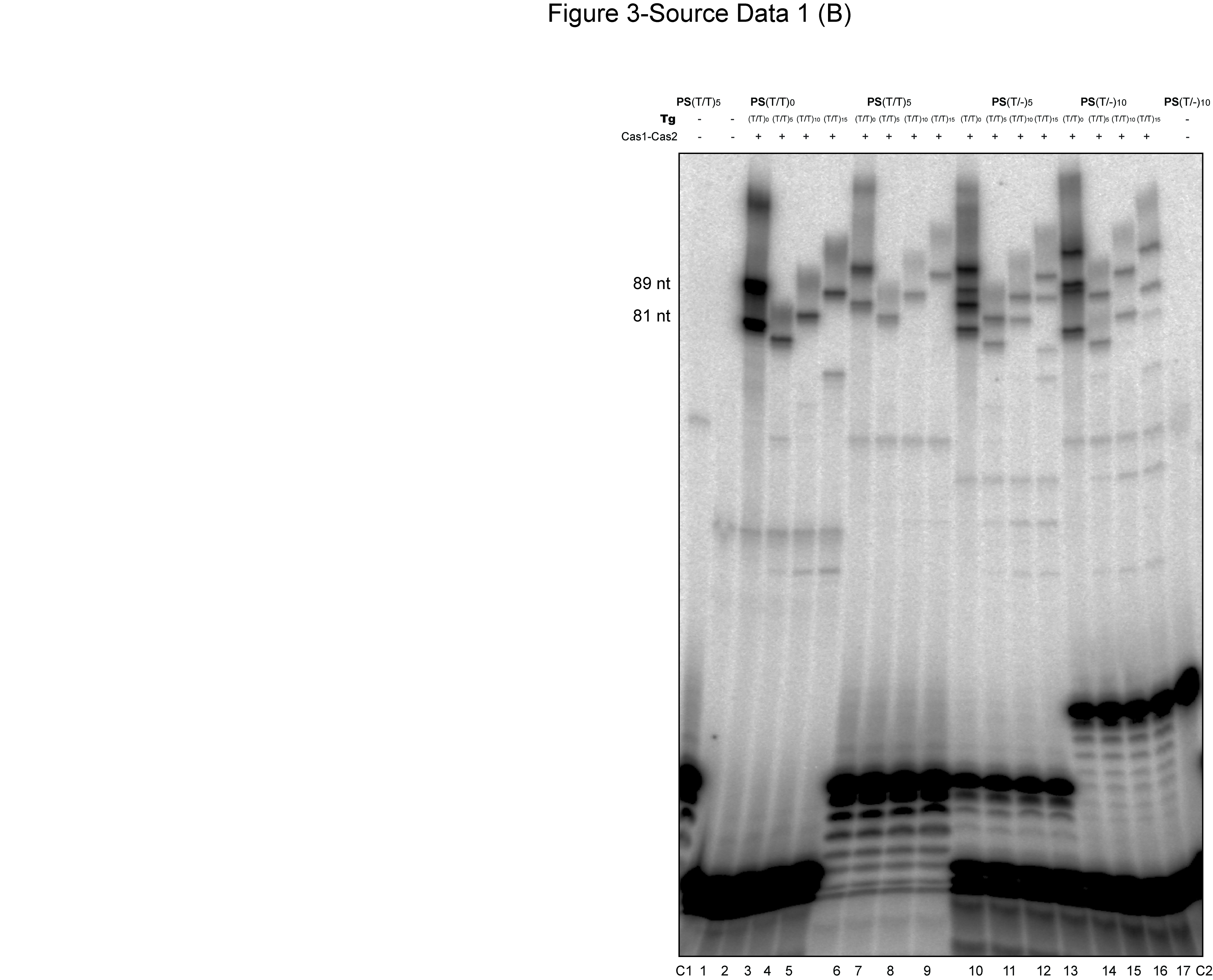

Supplement: Source data 2. [file elife-65763-data2.zip › CRISPR paper-Source Data-2/Figure 3- Source Data 1.tif]

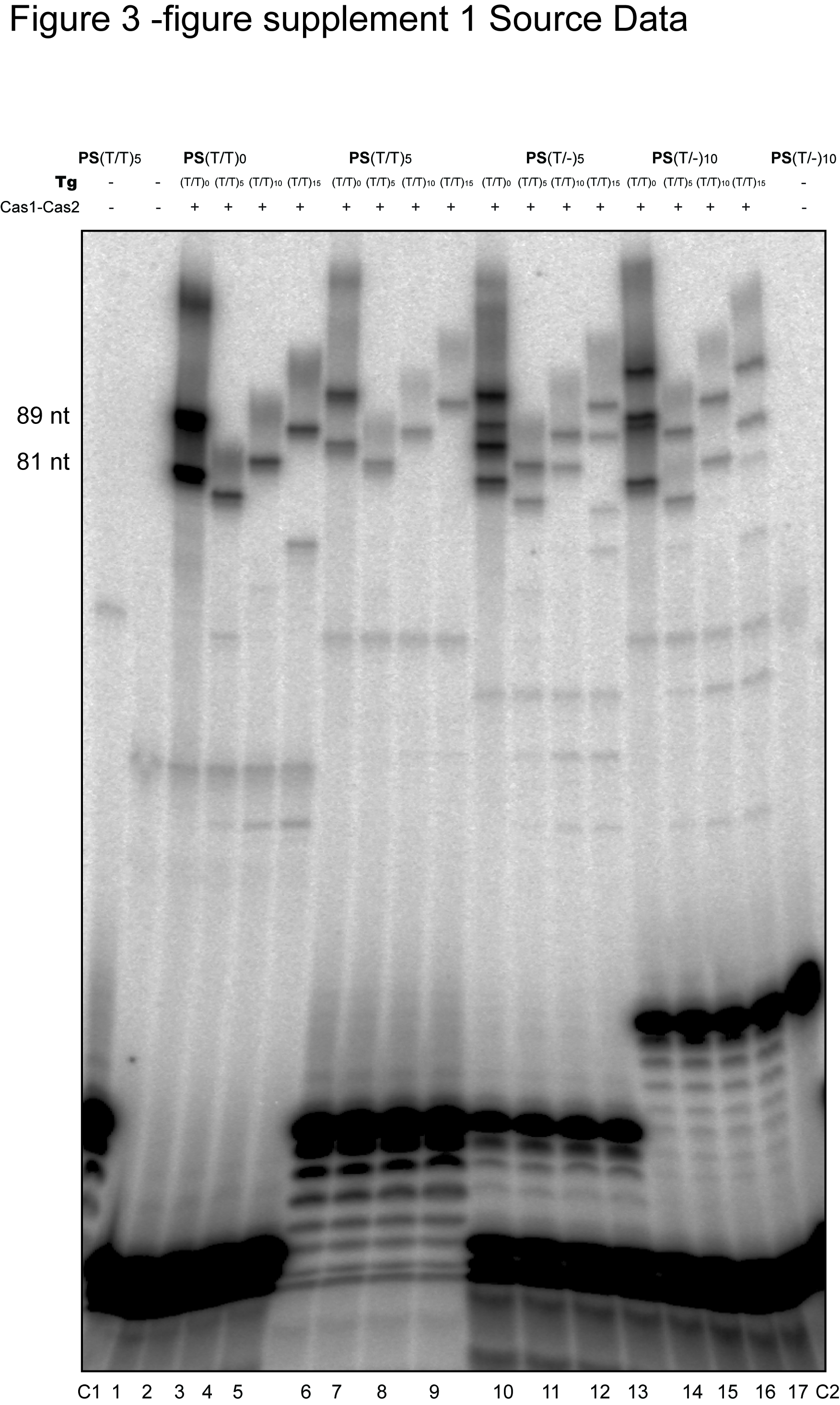

Supplement: Source data 2. [file elife-65763-data2.zip › CRISPR paper-Source Data-2/Figure 3-figure supplement 1 Source Data.tif]

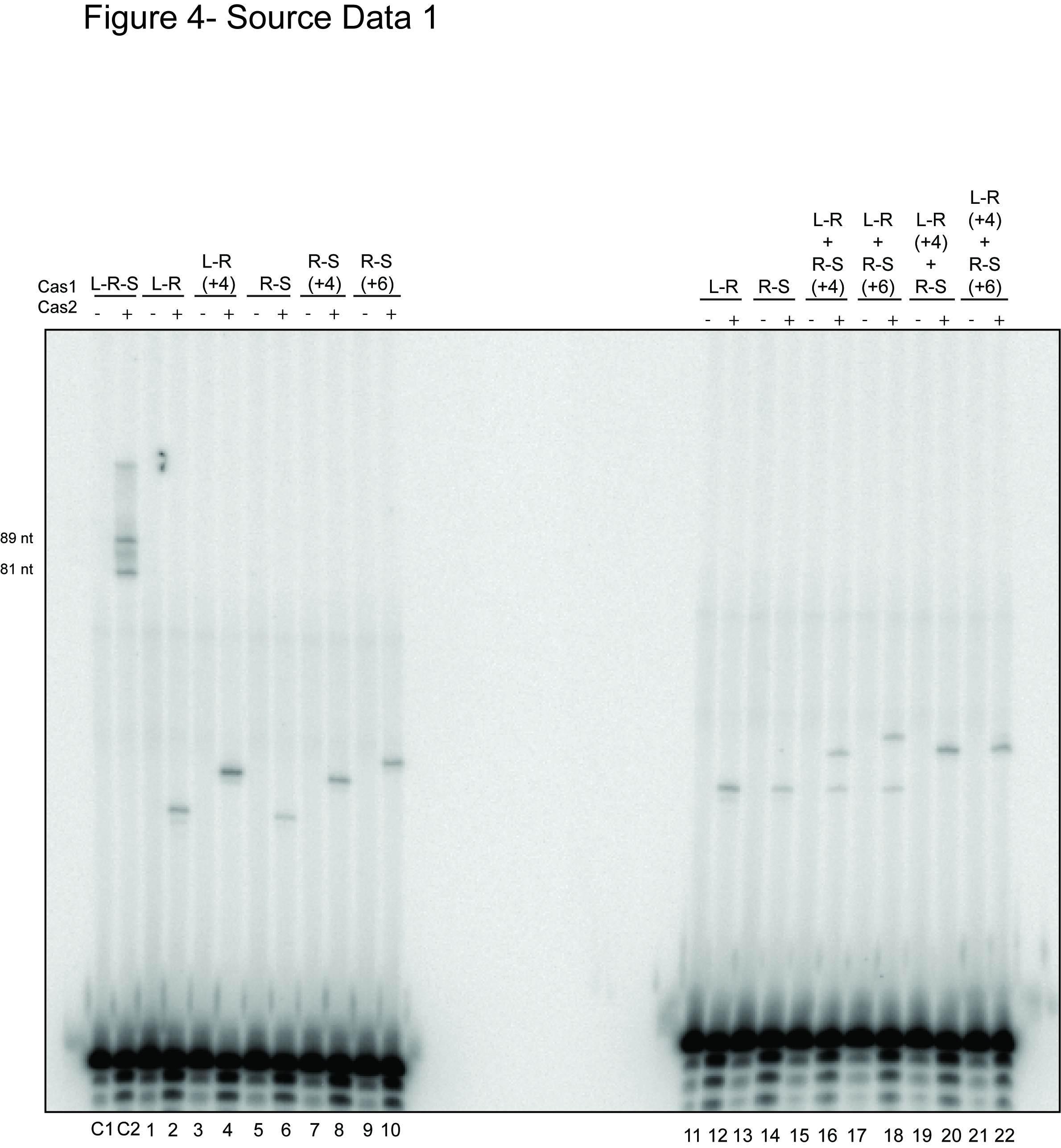

Supplement: Source data 2. [file elife-65763-data2.zip › CRISPR paper-Source Data-2/Figure 4- Source Data 1.tif]

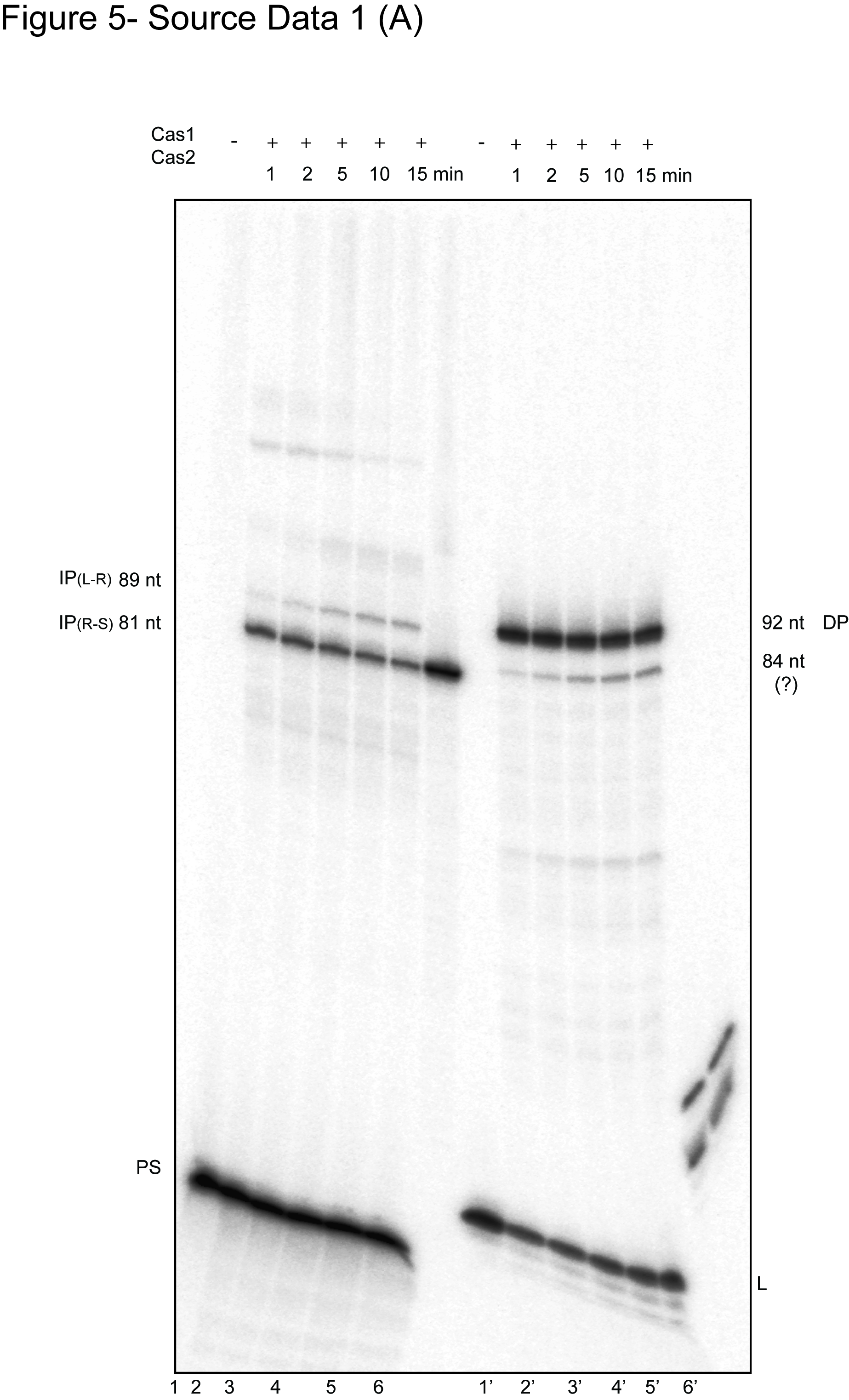

Supplement: Source data 2. [file elife-65763-data2.zip › CRISPR paper-Source Data-2/Figure 5- Source Data 1 (A).tif]

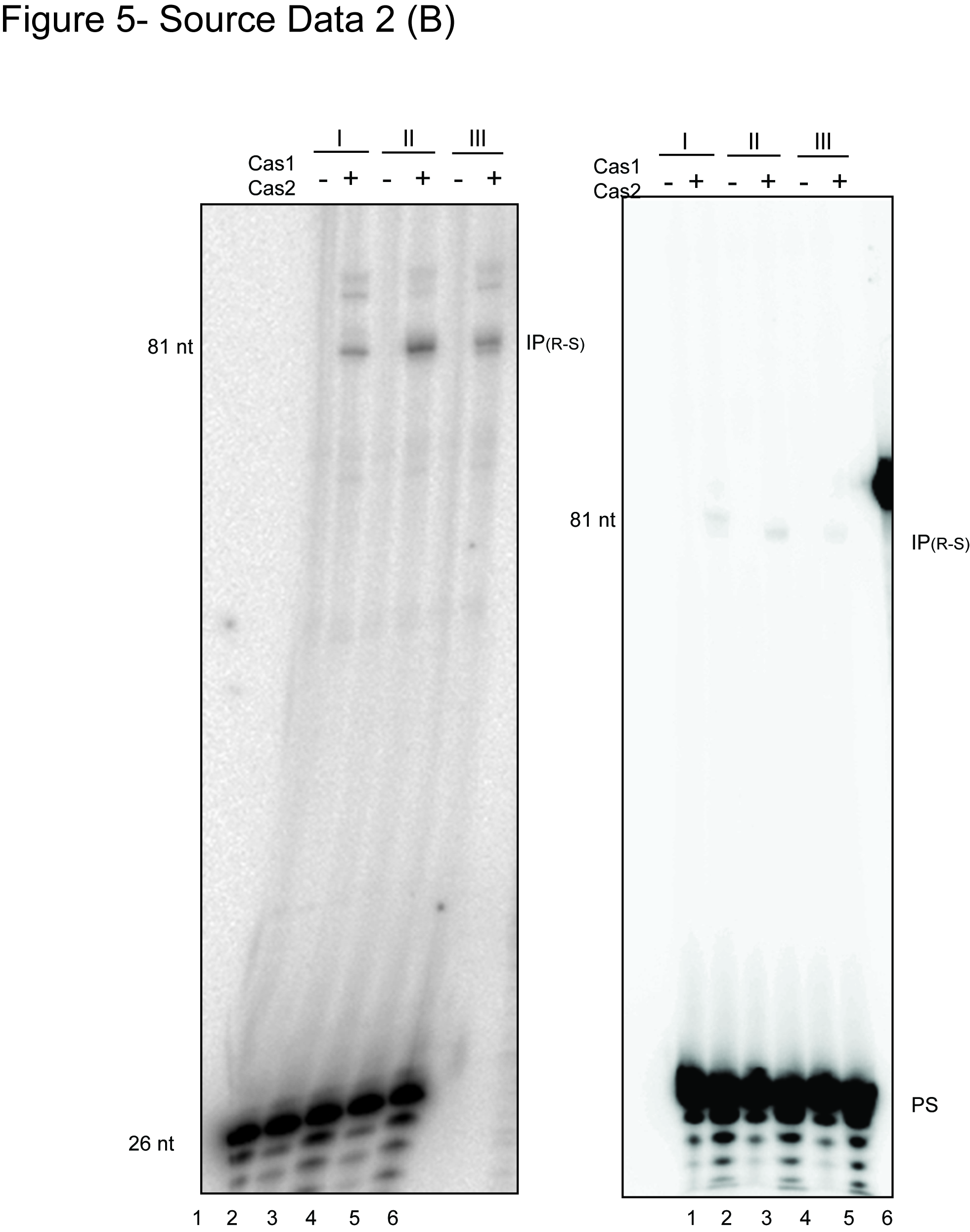

Supplement: Source data 2. [file elife-65763-data2.zip › CRISPR paper-Source Data-2/Figure 5- Source Data 2 (B).tif]

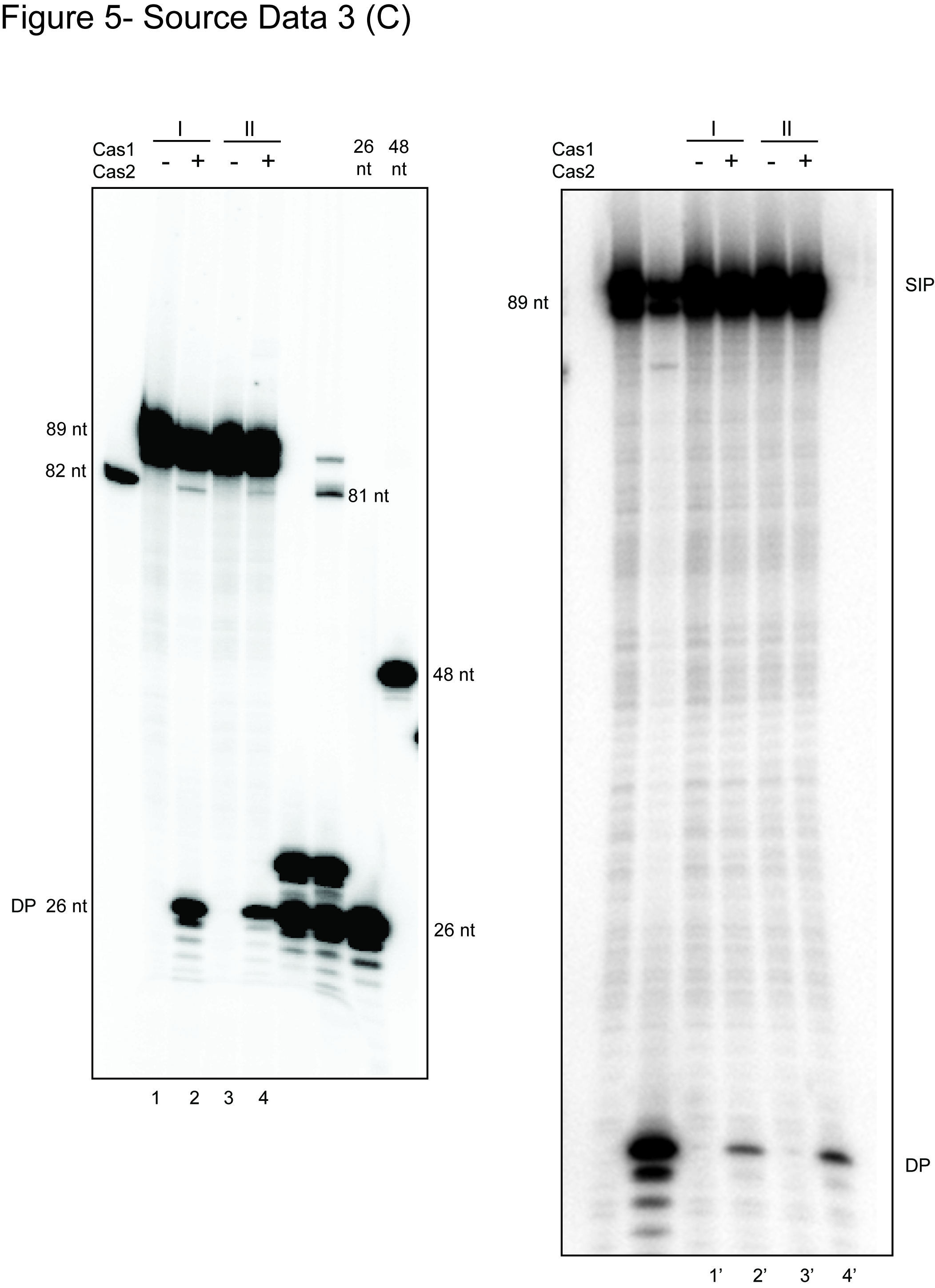

Supplement: Source data 2. [file elife-65763-data2.zip › CRISPR paper-Source Data-2/Figure 5- Source Data 3 (C).tif]

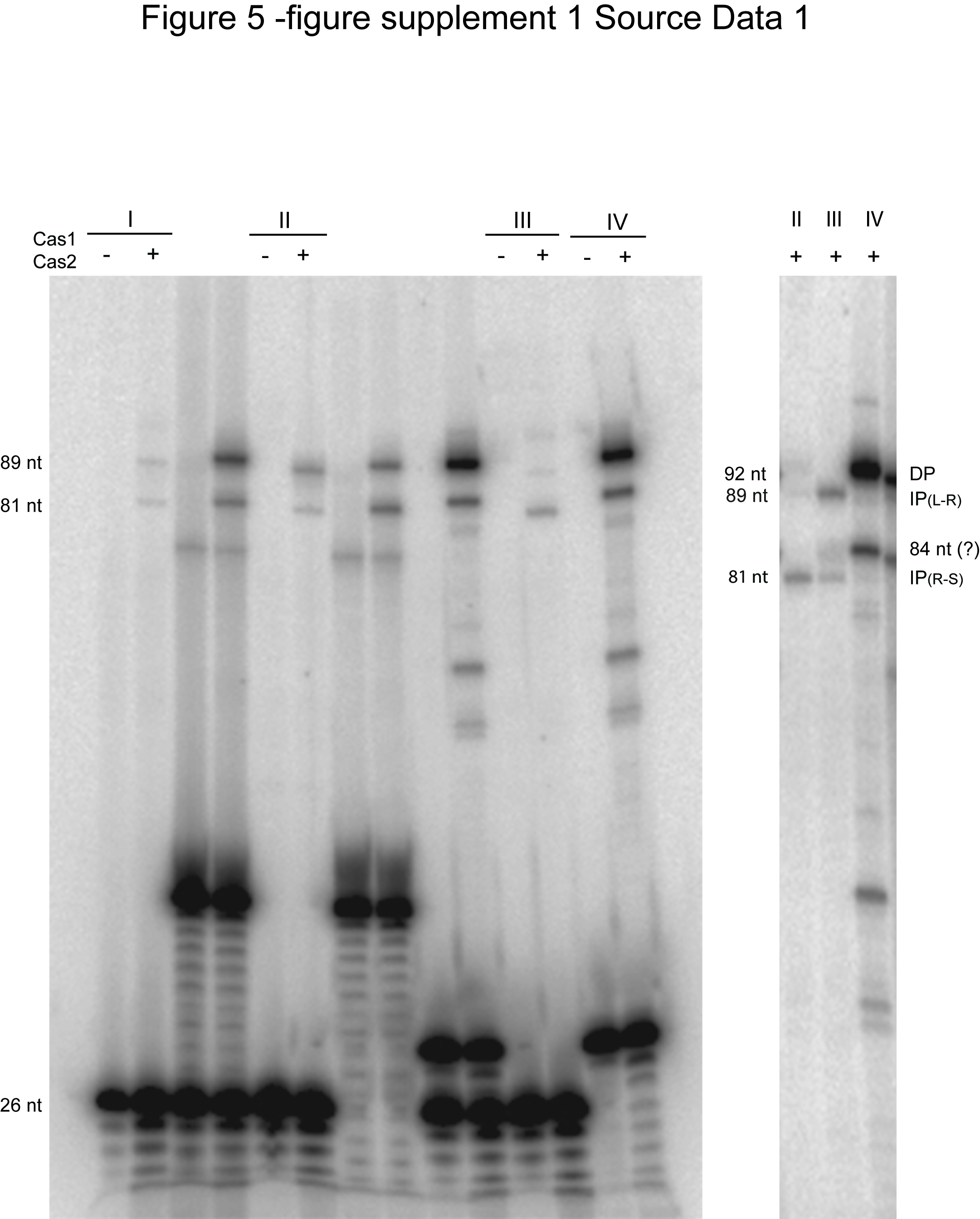

Supplement: Source data 2. [file elife-65763-data2.zip › CRISPR paper-Source Data-2/Figure 5-figure supplement 1 Source Data.tif]

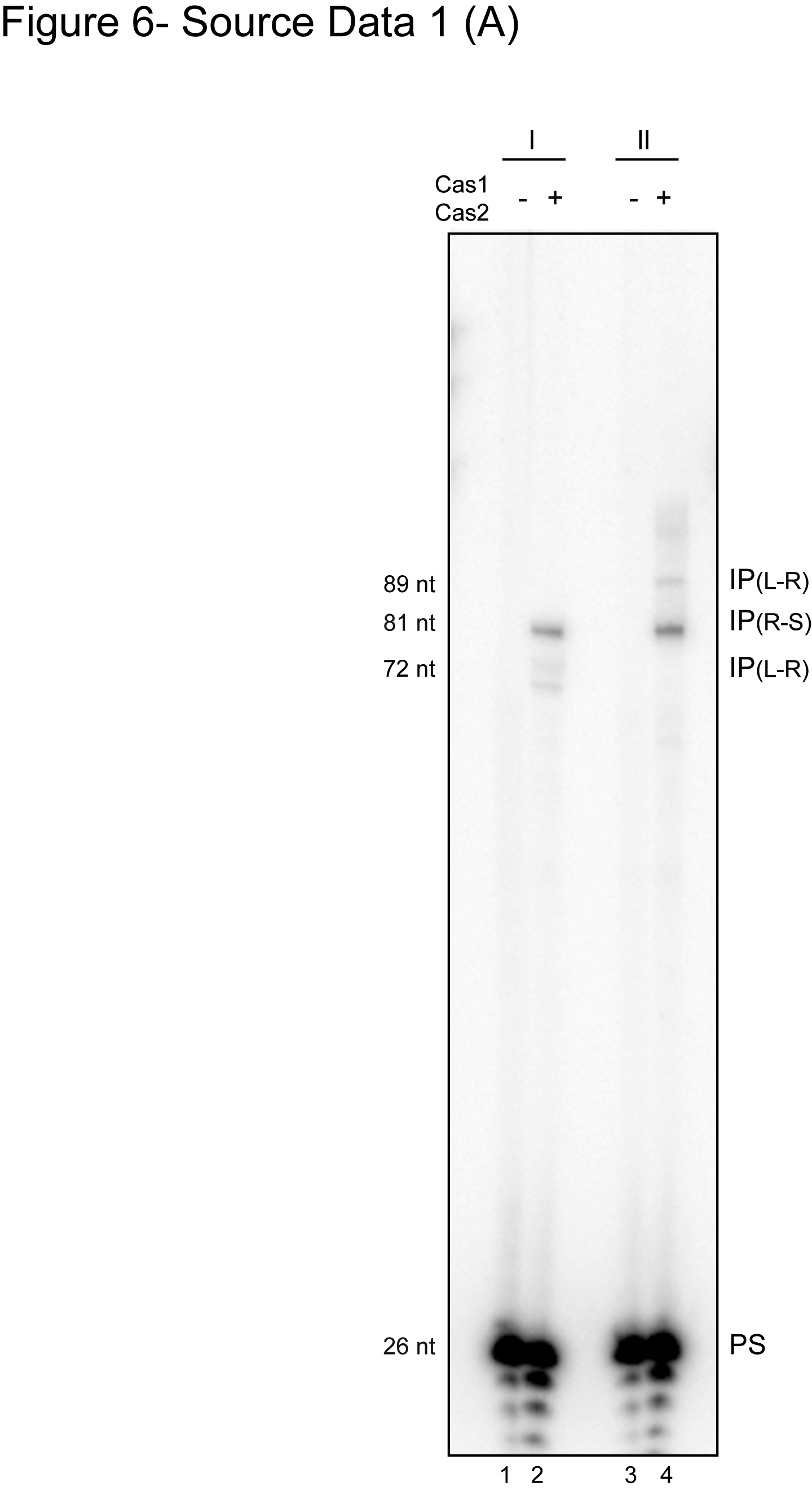

Supplement: Source data 2. [file elife-65763-data2.zip › CRISPR paper-Source Data-2/Figure 6- Source Data 1 (A).tif]

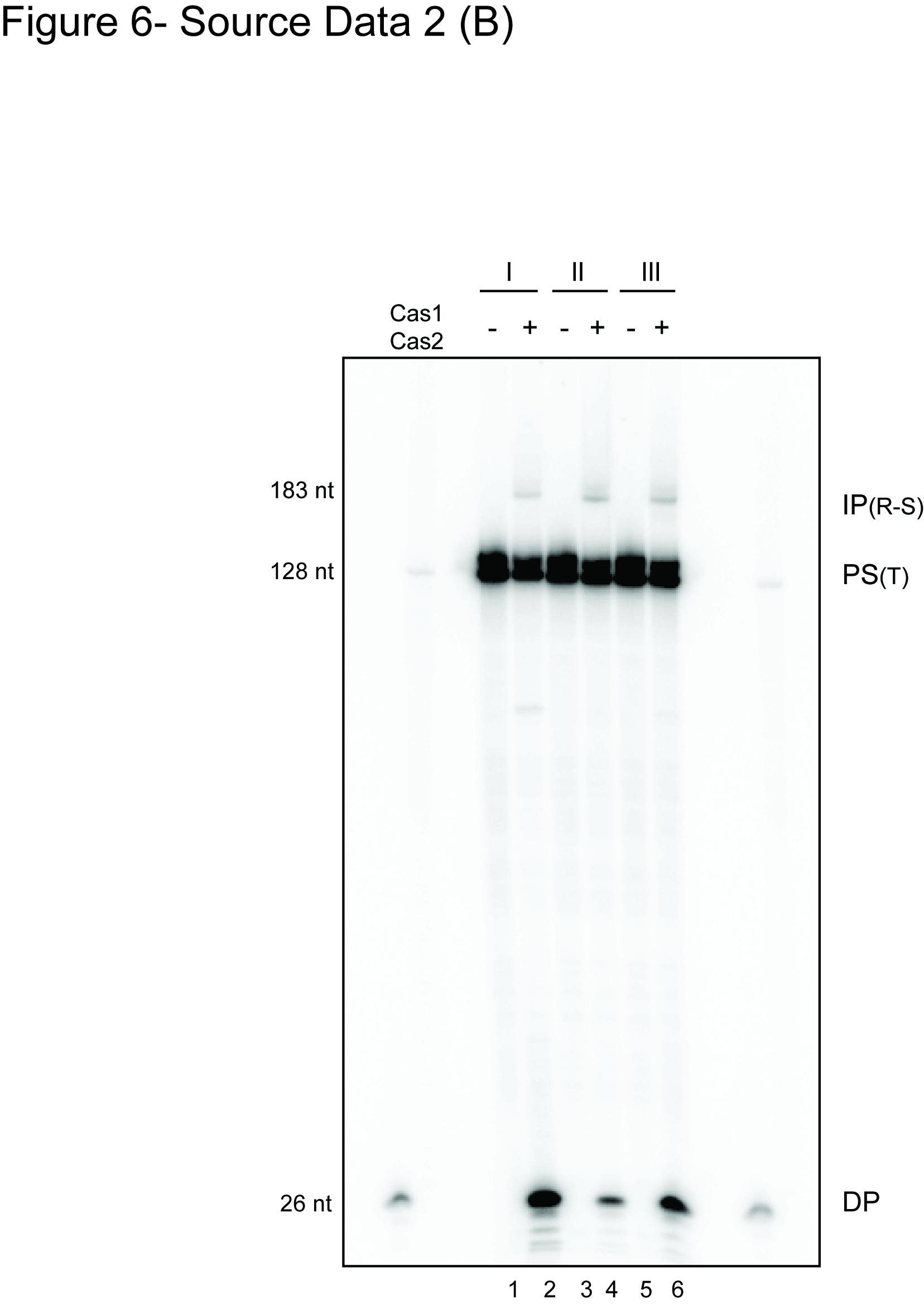

Supplement: Source data 2. [file elife-65763-data2.zip › CRISPR paper-Source Data-2/Figure 6- Source Data 2 (B).tif]

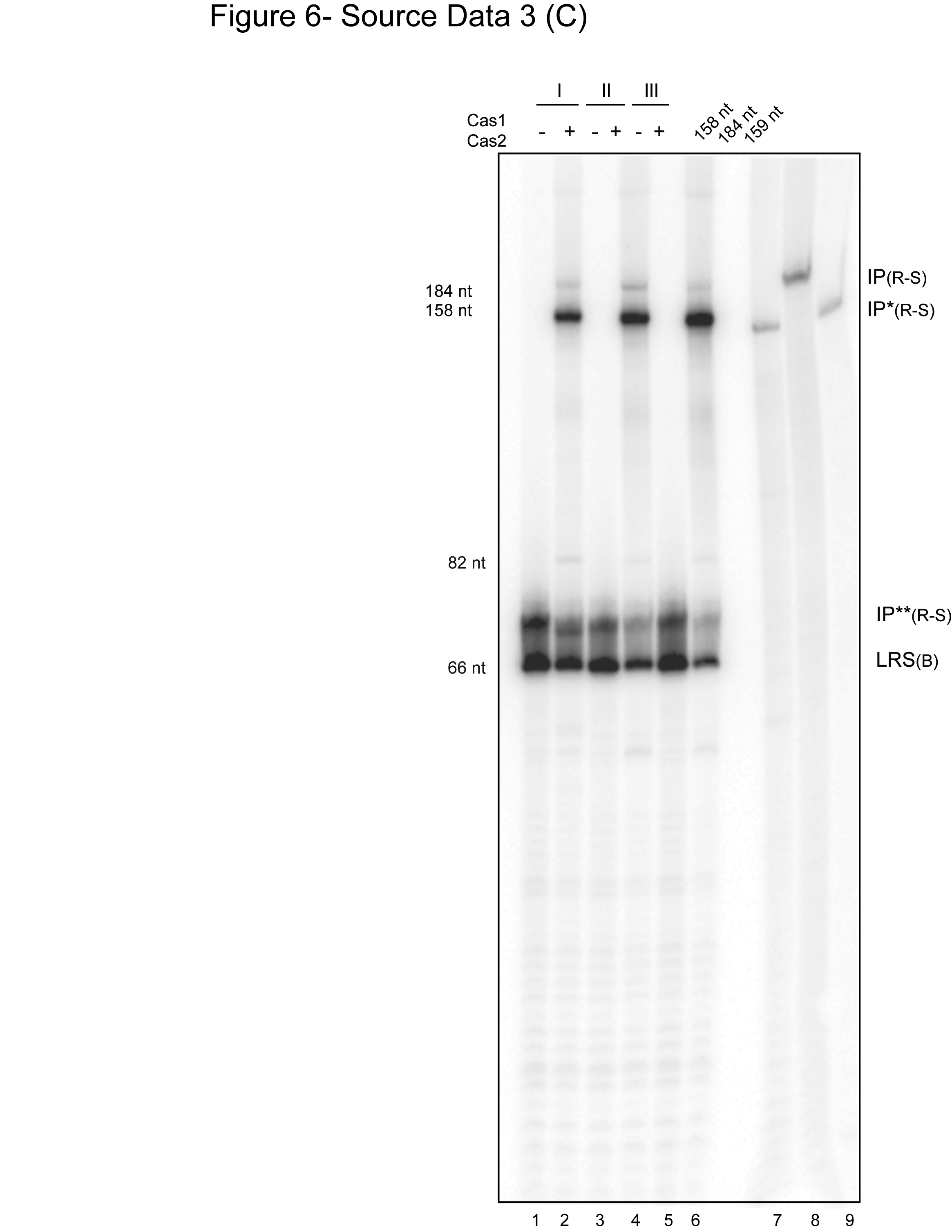

Supplement: Source data 2. [file elife-65763-data2.zip › CRISPR paper-Source Data-2/Figure 6- Source Data 3 (C).tif]
