# Supplementary material for: Disintegration promotes protospacer integration by the Cas1-Cas2 complex
Source: eLife. 2021 Aug 26;10:e65763. doi: 10.7554/eLife.65763 (PMC8390005; doi:10.7554/eLife.65763)
Supplement: Supplementary file 1. [file elife-65763-supp1.docx]

**Supplementary File 1**

| **Oligomer** | **Sequence (5’ to 3’)** | **Relevant figures** |
| --- | --- | --- |
| Proto-spacer | GAGTTACTACTCGTTCTGGCTCTGTC  gagccagaacgagtagtaactctgtc | Figures 1, 2, 3, 4, S1, S3, S4, S5, S7, S8 |
| Cy5-PS | Cy5-GAGTTACTACTCGTTCTGGCTCTGTC  Cy5-gagccagaacgagtagtaactctgtc | Figure S1 |
| Pre-spacer mimics | GAGTTACTACTCGTTCTGGCTCTGTCGGG  cccTTTTgagccagaacgagtagtaactctgtc  gagccagaacgagtagtaactctgtc | Figure S7 |
| Target site | CGATCGATTAGTCTACGAGGTTTTAGAGCTATGCTGTTTTGAATGGTCCCAAAACTGCGCTGGTTGATTTACATGTCTCTCT  agagagacatgtaaatcaaccagcgcagttttgggaccattcaaaacagcatagctctaaaacctcgtagactaatcgatcg | Figures 2, 3, 4, S4, S5, S7 and S8 |
| Target site (nicked) | CGATCGATTAGTCTAC  GAGGTTTTAGAGCTATGCTGTTTTGAATGGTCCCAAAACTGCGCTGGTTGATTTACATGTCTCTCT  AGAGAGACATGTAAATCAACCAGC  GCAGTTTTGGGACCATTCAAAACAGCATAGCTCTAAAACCTCGTAGACTAATCGATCG | Figures S4 |
| Proto-spacers with (T)n insertions | GAGTTACTACTtttttCGTTCTGGCTCTGTC  gagccagaacgtttttagtagtaactctgtc  GAGTTACTACTttttttttttCGTTCTGGCTCTGTC  GAGTTACTACTttttttttttCGTTCTGGCTCTGTC | Figures 3 and S5 |
| Target sites with (T)n insertions | TAGTCTACGAGGTTTTAGAGCtttttGTCCCAAAACTGCGCTGGTTGATTTACATGTCTCTCT  agagagacatgtaaatcaaccagcgcagttttgggactttttgctctaaaacctcgtagacta  TAGTCTACGAGGTTTTAGAGCttttttttttGTCCCAAAACTGCGCTGGTTGATTTACATGTCTCTCT  agagagacatgtaaatcaaccagcgcagttttgggacttttttttttgctctaaaacctcgtagacta  TAGTCTACGAGGTTTTAGAGCtttttttttttttttGTCCCAAAACTGCGCTGGTTGATTTACATGTCTCTCT  agagagacatgtaaatcaaccagcgcagttttgggactttttttttttttttgctctaaaacctcgtagacta | Figures 3 and S5 |
| Half-target site (L-R). | CGATCGATTAGTCTACGAGGTTTTAGAGCTATGCTGT  acagcatagctctaaaacctcgtagactaatcgatcg | Figure 4 |
| Half-target site (R-S) | TTTGAATGGTCCCAAAACTGCGCTGGTTGATTTACATGTCTCTCT  agagagacatgtaaatcaaccagcgcagttttgggaccattcaaa | Figure 4 |
| Half-target site (L-R, +4) | CGATCGATTAGTCTACGAGGTTTTAGAGCTATGCTGTtttg  caaaacagcatagctctaaaacctcgtagactaatcgatcg | Figure 4 |
| Half-target site (R-S, +4) | ctgtTTTGAATGGTCCCAAAACTGCGCTGGTTGATTTACATGTCTCTCT  agagagacatgtaaatcaaccagcgcagttttgggaccattcaaaacag | Figure 4 |
| Half-target site (R-S, +6) | tgctgtTTTGAATGGTCCCAAAACTGCGCTGGTTGATTTACATGTCTCTCT  agagagacatgtaaatcaaccagcgcagttttgggaccattcaaaacagca | Figure 4 |
| Semi-integration mimics | GAGTTACTACTCGTTCTGGCTCTGTCGTTTTAGAGCTATGCTGTTTTGAATGGTCCCAAAACTGCGCTGGTTGATTTACATGTCTCTCT  gagccagaacgagtagtaactctgtc  atcgatcgat CGATCGATTAGTCTACGAG  agagagacatgtaaatcaaccagcgcagttttgggaccattcaaa  acagcatagctctaaaacctcgtagactaatcgatcg  atcgatcgatCGATCGATTAGTCTACGAC/3ddC  GAGTTACTACTCGTTCTGGCTCTGTCGTTTTAGAGCTATGCTGTTTTGAATGGTCCCAAAACTGCGCTGGTT  aaccagcgcagttttgggaccattcaaaacagcatagctctaaaacctcgtagactaatcgatcg  GAGTTACTACTCGTTCTGGCTCTGTCGTTTTAGAGCTATGCTGTTTTGAATGGTCCCAAAACTGCGCTGGTTtttttctctcttttttGagccagaacgagtagtaactctgtc | Figures 5, 6 and S8  Figures 5 and 6  Figure 6 |
| Semi-integration mimics | GAGTTACTACTCGTTCTGGCTCTGTCgttttgggaccattcaaaacagcatagctctaaaacctcgtagactaatcgatcg  gagccagaacgagtagtaactctgtc  atcgatcgatagagagacatgtaaatcaaccagcgca  CGATCGATTAGTCTACGAGGTTTTAGAGCTATGCTGTTTTGAATGGTCCCAAAACTGCGCTGGTTGATTTACATGTCTCTCT | Figure S8 |

**Supplementary File 1. Synthetic oligonucleotides**. The sequences of the oligonucleotides used for this study are listed. The lower case ‘t’s refer to thymine insertions that remain bulged (with no paired or unpaired bases on the opposite strand) or double-looped (unpaired thymine bases on the opposite strand) in the assembled substrates. The ‘dd’ abbreviation refers to ‘dideoxy’. The assays in which the oligos were used are indicated by the corresponding figure numbers.
